# Multisample Mass Spectrometry-Based Approach for Discovering Injury Markers in Chronic Kidney Disease

**DOI:** 10.1074/mcp.RA120.002159

**Published:** 2021-01-13

**Authors:** Ji Eun Kim, Dohyun Han, Jin Seon Jeong, Jong Joo Moon, Hyun Kyung Moon, Sunhwa Lee, Yong Chul Kim, Kyung Don Yoo, Jae Wook Lee, Dong Ki Kim, Young Joo Kwon, Yon Su Kim, Seung Hee Yang

**Affiliations:** 1 Department of Internal Medicine, Seoul National University Hospital, Seoul, Korea; 2 Department of Internal Medicine, Korea University Guro Hospital, Seoul, Korea; 3 Proteomics Core Facility, Seoul National University Hospital, Seoul, Korea; 4 Biomedical Research Institute, Seoul National University Hospital, Seoul, Korea; 5 Department of Internal Medicine, Veterans Health Service Medical Center, Seoul, Korea; 6 Department of Internal Medicine, Kangwon National University Hospital, Gangwon-Do, Korea; 7 Department of Internal Medicine, Ulsan University Hospital, Ulsan, Korea; 8 Nephrology Clinic, National Cancer Center, Goyang, Gyeonggi-do, Korea; 9 Kidney Research Institute, Seoul National University College of Medicine, Seoul, Korea

**Keywords:** chronic kidney disease, mass spectrometry, proteomics, fibrosis, apoptosis, inflammation, LGALS-1, CKD, chronic kidney disease, DEP, differentially expressed protein, ECM, extracellular matrix, FASP, filter-aided sample preparation, FDR, false discovery rate, GEC, glomerular endothelial cell, GO, gene ontology, MS, mass spectrometry, PBS, phosphate-buffered saline, PCA, principal component analysis, PLS-DA, partial least squares–discriminant analysis, PTEC, proximal tubular epithelial cell, SDS, sodium dodecyl sulfate, TMT, tandem mass tag

## Abstract

Urinary proteomics studies have primarily focused on identifying markers of chronic kidney disease (CKD) progression. Here, we aimed to determine urinary markers of CKD renal parenchymal injury through proteomics analysis in animal kidney tissues and cells and in the urine of patients with CKD. Label-free quantitative proteomics analysis based on liquid chromatography–tandem mass spectrometry was performed on urine samples obtained from 6 normal controls and 9, 11, and 10 patients with CKD stages 1, 3, and 5, respectively, and on kidney tissue samples from a rat CKD model by 5/6 nephrectomy. Tandem mass tag-based quantitative proteomics analysis was performed for glomerular endothelial cells (GECs) and proximal tubular epithelial cells (PTECs) before and after inducing 24-h hypoxia injury. Upon hierarchical clustering, out of 858 differentially expressed proteins (DEPs) in the urine of CKD patients, the levels of 416 decreased and 403 increased sequentially according to the disease stage, respectively. Among 2965 DEPs across 5/6 nephrectomized and sham-operated rat kidney tissues, 86 DEPs showed same expression patterns in the urine and kidney tissue. After cross-validation with two external animal proteome data sets, 38 DEPs were organized; only ten DEPs, including serotransferrin, gelsolin, poly ADP-ribose polymerase 1, neuroblast differentiation-associated protein AHNAK, microtubule-associated protein 4, galectin-1, protein S, thymosin beta-4, myristoylated alanine-rich C-kinase substrate, and vimentin, were finalized by screening human GECs and PTECs data. Among these ten potential candidates for universal CKD marker, validation analyses for protein S and galectin-1 were conducted. Galectin-1 was observed to have a significant inverse correlation with renal function as well as higher expression in glomerulus with chronic injury than protein S. This constitutes the first multisample proteomics study for identifying key renal-expressed proteins associated with CKD progression. The discovered proteins represent potential markers of chronic renal cell and tissue damage and candidate contributors to CKD pathophysiology.

## Highlights

• This study aimed to specify chronic kidney injury markers through proteomics
• Ten putative candidate proteins were found to increase with chronic kidney injury
• Galectin-1 and protein S were elevated in glomeruli and tubules after chronic injury
• Galectin-1 also showed an inverse correlation with renal function

## Graphical Abstract

**Figure:**
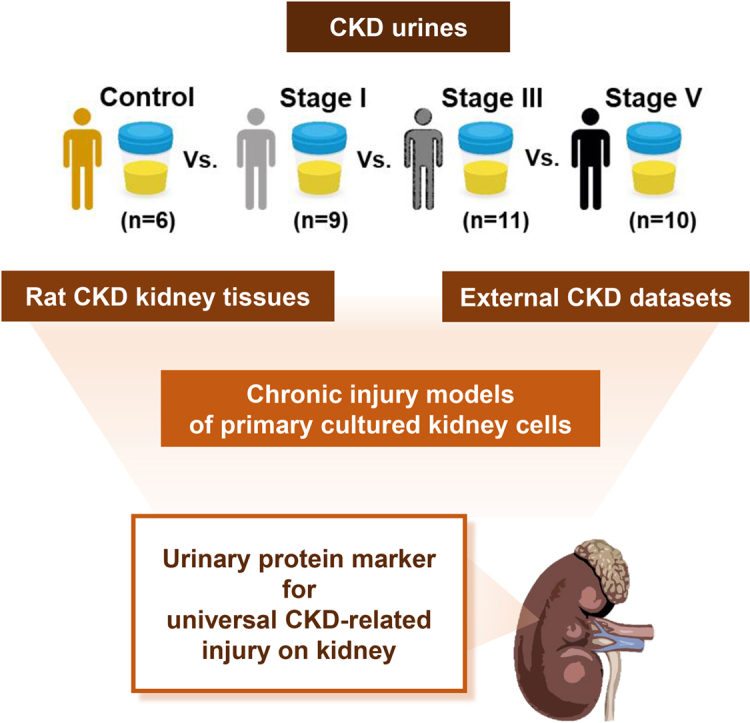

## In Brief

The key renal-expressed proteins including protein S and galectin-1 associated with CKD stages were determined by applying quantitative MS-based proteomics using multiple types of samples. The discovered proteins represent potential markers of chronic kidney injury related to renal hypoxia and candidate contributors to CKD pathophysiology.

Chronic kidney disease (CKD) is characterized by a slow and gradual loss of kidney function, with glomerular filtration loss over months or years, inevitably leading to end-stage renal disease (1). The renal failure resulting from this irreversible process arises from fibrotic lesions, including glomerulosclerosis, vascular sclerosis, and tubulointerstitial fibrosis, of each compartment of the kidney (2, 3). Nevertheless, despite numerous research efforts, both the definitive mechanism underlying the progression from CKD to end-stage renal disease and an effective treatment have remained elusive.

Recently, as various multiomics approaches have been spotlighted, proteomics analyses have been attempted for predicting the progression of CKD. Over the past decade, proteomics for CKD has focused on the development of noninvasive markers for CKD progression based on urinary proteins (4, 5, 6). However, urinary proteins and peptides originate not only from glomerular filtration but also from tubular secretion, epithelial cells shed from the kidney and urinary tract, and secreted exosomes. Thus, whereas urinary protein differences may represent a suitable marker for overall renal failure, they do not specifically reflect renal parenchymal injury.

Mass spectrometry (MS)-based proteomics has developed to be an appropriate tool for systematic analysis to identify and quantify thousands of proteins (7, 8). Accordingly, numerous different quantitation strategies are now commonly used in proteomics experiments (9, 10, 11, 12, 13). For example, label-free techniques are used to measure protein abundance based on peptide ion intensities or spectral counts (9, 14), and mass-difference labeling methods by tandem mass tag (TMT) allow binary or tertiary comparisons to be made within the same experiment during MS analysis (13, 15).

In this study, we therefore aimed to identify specific proteins involved in the various processes of inflammation, apoptosis, and fibrosis of damaged renal parenchyma *via* multiple proteomics analyses. We utilized both label-free and TMT-multiplexed quantitative proteomics approaches to identify protein expression changes associated with chronic injury in three types of specimens including primary cultured renal cells, rat kidney tissues, and human urine samples obtained from patients with CKD. Using this strategy, we identified multiple urinary marker candidates associated with universal injury in CKD.

## Experimental Procedures

### Experimental Design and Statistical Rationale

The three types of samples used for multisample proteomic analysis in this study are as follows:

The urine samples were obtained from 6 healthy controls and 9, 11, and 10 patients with CKD stages 1, 3, and 5, respectively. The rat kidney tissues were obtained from five animals for each of sham-operated group and 5/6 nephrectomy group. Human primary glomerular epithelial cells (GECs) and primary proximal tubular epithelial cells (PTECs) were respectively cultured in three separate experiments under two conditions, hypoxia and normoxia. These repeated individual experiments were conducted to achieve adequate reliability and consistency in quantitative proteomic profiling.

### Collection of Urine Samples From Patients With CKD

The Institutional Review Board of the Seoul National University Hospital reviewed and approved the protocols for obtaining urine samples from the patients who provided written informed consent (IRB number: 1710-058-894). CKD was diagnosed and classified according to Kidney Disease: Improving Global Outcomes (KDIGO) guidelines based on estimated glomerular filtration rates (eGFR) (1). For urinary proteomics analysis, urine samples of the healthy controls and the patients with CKD stages 1, 3, and 5 were collected. All urine samples were collected randomly as a midstream, clean catch specimen into a sterile urine container and immediately transported on ice. The samples were then clarified, and insoluble materials were removed by centrifugation at 2000*g* at 4 °C for 10 min. Supernatants were collected and stored at −80 °C until usage. Demographic characteristics including age and sex and laboratory results including blood urea nitrogen, creatinine, eGFR, and urine protein creatinine ratio were collected from the electronic medical records.

### Rat CKD Model

All experiments were performed in accordance with the Guidelines for the Care and Use of Laboratory Animals of the National Research Council and the U.S. National Institutes of Health under the approval of the Institutional Animal Care and Use Committee of the Clinical Research Institute at Seoul National University Hospital (Approval number: 18-0222-S1A0). A rat CKD model was developed using a modified protocol described as follows (16). Male Sprague-Dawley rats weighing 160 to 200 g were purchased from the Jackson Laboratory. The 5/6 nephrectomy was performed under anesthesia with a mixture of ketamine (100 mg/ml) and xylazine (25 mg/ml). Briefly, the resection of two-thirds of the right kidney was performed and then the left nephrectomy was performed after 1 week. The time of left nephrectomy marked the onset of moderate-to-severe renal failure.

For analysis of renal function, serum blood urea nitrogen and creatinine were measured in blood samples from the animals, and then the animals were subjected to a 24-h urine collection to determine protein and creatinine concentration and urine volume. In addition, blood pressure was measured using the tail-cuff method (CODA system, Kent Scientific Corporation). Mice were sacrificed at the end of the experiments, and the kidneys were harvested.

### Primary GEC and PTEC Culture and Induction of Hypoxic Injury

We isolated GECs and PTECs from normal adjacent kidney tissue from patients with renal cell carcinoma according to guidelines approved by the Institutional Review Board of Seoul National University Hospital (IRB no. 1404-117-515) (17). The renal cortex was dissected from the kidney sections obtained from nephrectomy specimens. Then, the dissected tissues were digested with Hanks balanced salt solution containing 1% FBS and 3 mg/ml collagenase (Sigma-Aldrich) at 37 °C for 1 h. The digested cortices were sifted through several sizes of sieves (150, 120, 90 μm) and centrifuged twice at 500*g* for 5 min and then incubated in DMEM/F12 (Lonza).

For isolation of tubular cells, after 4 to 6 h of incubation, floating tubular cells were collected and cultured on collagen-coated dishes (BD Biosciences) until colonies were established. After 10 days, cultured PTECs were analyzed and isolated by a fluorescence-activated Cell Sorting (FACS) Calibur instrument (BD Biosciences) with staining by FITC-labeled anti-AQP1 (Abcam) at 4 °C for 30 min.

For GECs, isolated glomeruli were cultured for 8 to 10 days. After the outgrowing cells were trypsinized and passed through serial sieves, the cells were washed twice with phosphate-buffered saline (PBS). The cultured cells were incubated with Fc receptor blocking reagent (1 μg/ml, BD Bioscience). Glomerular endothelial cells were identified using PE-conjugated anti-CD31 (BD Bioscience). Stained cells were sorted and analyzed using an fluorescence-activated Cell Sorting Calibur instrument (BD Biosciences). The cells were cultured with medium consisting of RPMI-1640 (Sigma-Aldrich) supplemented with 20% heat-inactivated fetal bovine serum, 5  ng/ml vascular endothelial growth factor, 10 ng/ml basic fibroblast growth factor, 10  ng/ml epidermal growth factor, 20 U/ml heparin, 1 mg/ml hydrocortisone, 50 U/ml penicillin, and 50 mg/ml streptomycin. After 10 days of culture, cells were detached from the dishes by the addition of 3 mM EDTA solution and a minimal amount of trypsin. Cells (2 × 10^5^/well) were then placed in 6-well chamber slides with serum-free medium for 24 h and washed twice with PBS. Cells were incubated under hypoxic (1% O_2_) or normoxic conditions (20% O_2_) for 24 h.

### Sample Preparation for MS Analysis

For relative quantification of human urine and rat kidney tissue before and after CKD progression, we performed label-free quantitation. CKD urine samples were centrifuged for 15 min at 3000*g* to remove debris. Urine supernatants were concentrated using an Amicon Ultra centrifugal filter device (3 kDa MWCO, Millipore) at 14,000*g* to a volume of approximately 50 μl. The protein content of the final concentrated solution was determined using the Bradford method (Bio-Rad Protein Assay, Bio-Rad). For label-free quantification, 100 μg of urine proteins was precipitated by adding a sixfold volume of ice-cold acetone prior to the digestion step. Precipitated proteins were dissolved in sodium dodecyl sulfate (SDS)-containing denaturation buffer. After being heated at 99 °C, the denatured proteins were digested using a modified filter-aided sample preparation (FASP) method (18, 19). The proteins were digested with trypsin (1:100 enzyme-to-protein ratio [w/w]) at 37 °C overnight.

Kidney tissue samples were prepared using the FASP method modified for frozen tissue preparation (20). First, frozen tissue samples were homogenized with lysis buffer (4% SDS, 2 mM tris(2-carboxyethyl)phosphine, and 0.1 M Tris-HCl, pH 7.4). The protein concentration was determined using a reducing agent-compatible BCA assay kit (Thermo Fisher Scientific). To remove contaminants, we performed acetone precipitation at −20 °C with 200 μg of the lysate. The protein pellet was dissolved in 50 μl of reduction buffer (4% SDS, 0.1 mM dithiothreitol, and 0.1 M Tris-Cl, pH 7.4) and heated at 95 °C for 15 min. Reduced proteins were loaded onto a 30 K spin filter (Millipore). Buffer was exchanged with UA solution (8 M urea in 0.1 M Tris-Cl, pH 8.5) by centrifugation. After triple UA exchange, the reduced cysteines were alkylated with 0.05 M iodoacetamide in UA solution for 30 min at room temperature in the dark. We replaced the UA buffer with 40 mM ammonium bicarbonate followed by digestion with trypsin (1:100 enzyme-to-substrate ratio) at 37 °C for 16 h. Then, the digested peptides were collected by centrifugation and an additional elution step was subsequently performed with 40 mM ammonium bicarbonate and 0.5 M NaCl. All samples were desalted and fractionated using homemade C18-SDB-StageTips as previously described (21).

For relative quantification of human primary GECs before and after hypoxic injury, we performed TMT-labeled MS analysis. Proteins extracted from primary cells were digested by the FASP method as described above. Primary cell pellets were lysed in lysis buffer (4% SDS, 2 mM tris(2-carboxyethyl)phosphine, and 0.1 M Tris-HCl, pH 7.5) by direct sonication. Lysates were heated for 30 min at 95 °C. To minimize interference from the reducing reagent, the concentration of proteins in lysates was measured using a reducing-agent-compatible BCA assay (Thermo Fisher Scientific). After each sample containing 150 μg of total protein was precipitated with cold acetone, the proteins were digested *via* multidigestion FASP according to our previously described process (20). Peptide concentrations were measured by tryptophan fluorescence assay (22). TMT labeling was performed according to the manufacturer’s protocol with some modifications (23). Briefly, the TMT reagent (0.8 mg) was dissolved in 100% acetonitrile. After spiking with 500 ng of peptides derived from ovalbumin as an internal standard, 25 μl of the reagent was added to 50 μg of peptide samples along with acetonitrile to give a final concentration of 30% (v/v). Normoxia samples were labeled with the tags TMT-126, TMT-127, and TMT-128, whereas hypoxia samples were labeled with tags TMT-129, TMT-130, and TMT-131. After incubation at room temperature for 1 h, the reaction was quenched with 15.3 μl of 5% hydroxylamine. TMT-labeled samples were pooled at a 1:1:1:1:1:1 ratio. The resulting peptide mixtures were vacuum-centrifuged to dry and subjected to C18 solid-phase extraction. The pooled peptides were subjected to high pH reverse-phase-high-performance liquid chromatography (HPLC) fractionation using an Agilent 1290 bioinert HPLC (Agilent) equipped with an analytical column (4.6 × 250 mm, 5 μm) as described previously (20). Solvent A consisted of 15 mM ammonium hydroxide in water and solvent B consisted of 15 mM ammonium hydroxide in 90% acetonitrile. The peptides were separated with a 5 to 35% acetonitrile gradient at a flow rate of 0.2 ml/min. A total of 96 fractions were concatenated to mix different parts of the gradient into 24 fractions. The fractions were lyophilized and stored at −80 °C until MS analysis.

### Liquid Chromatography–Tandem MS (LC-MS/MS) Analysis

LC-MS/MS analysis was performed using a Q-Exactive HF-X (label-free quantification) or a Q-Exactive plus (TMT 6-plex experiments) Quadrupole-Orbitrap mass spectrometer (Thermo Fisher Scientific) coupled to an Ultimate 3000 RSLC system (Dionex) equipped with an EASY nanospray source (Thermo Fisher Scientific) as previously described (20, 24). For label-free quantification, peptide samples were loaded onto a trap column and separated on an analytic column (75 μm × 50 cm) with a 90-min gradient from 10 to 30% acetonitrile at 300 nl/min. For TMT, labeled peptide samples were separated on a two-column system consisting of a trap column and an analytic column (75 μm × 50 cm) with a 180-min gradient from 10 to 30% acetonitrile at 300 nl/min and analyzed by MS. Column temperature was maintained at 60 °C using a column heater. For label-free quantification, the MS scanned a mass range of 350 to 1650 m/z with a resolution of 70,000 at m/z 200 for precursor ions. A top-15 method was used to select precursor ions with an isolation window of 0.7 m/z. MS/MS spectra were acquired at a higher-energy collisional dissociation-normalized collision energy of 30, with a resolution of 17,500, at m/z 200. In case of TMT 6-plex, survey scans were acquired with a resolution of 70,000 at m/z 200. A top-15 method was used to select the precursor ion with an isolation window of 0.7 m/z. The MS/MS spectrum was acquired at a higher-energy collisional dissociation-normalized collision energy of 32 with a resolution of 35,000 at m/z 200. The maximum ion injection times for the full scan and MS/MS scan were 20 and 120 ms, respectively

### Data Processing for MS Analysis

For label-free quantification, MS raw files for CKD urine and rat kidney tissue were analyzed in a MaxQuant (version 1.6.1.0) environment (25). Mass tolerance was set to 6 ppm for monoisotopic precursor ions and 20 ppm for MS/MS peaks. Enzyme specificity was set to trypsin/P and a maximum of two missed cleavages was allowed. A minimal peptide length of six amino acids was required. Cysteine carbamidomethylation and methionine oxidation were considered as fixed and variable modifications, respectively. The spectra were searched using the Andromeda search engine against the human UniProt sequence database (December 2014, 88,657 entries) for CKD urine or the RAT UniProt sequence database (September 2018, 37,316 entries) for rat kidney tissue with 248 common contaminants and concatenated with the reversed versions of all sequences. The false discovery rate (FDR) was set to 1% for peptide and protein identifications. The peptide identifications across different LC-MS runs and the spectral library were matched by enabling the “match between runs” feature in MaxQuant. Contaminants, reverse, and only site identifications were excluded from further data analysis. Protein quantification was based on the intensity-based absolute quantification (iBAQ) algorithm integrated into the MaxQuant platform (26).

For the database search for the TMT 6-plex experiment, MS raw files were processed using Proteome Discoverer version 2.2 with the SEQUEST-HT algorithm against the human UniProt protein sequence database (December 2014, 88,657 entries). The search parameters included full enzyme digestion using trypsin with up to two missed cleavages, 20 ppm of peptide precursor mass tolerance, and 0.02 Da of fragment ion mass tolerance. Variable modifications of 15.995 Da for methionine oxidation and 42.011 Da for protein N-terminal acetylation and fixed modifications of 57.021 Da for carbamidomethylation on cysteine residues and 229.153 Da for TMT 6-plex labeled lysine and any N-terminus were selected. The coisolation threshold for quantification of peptides was set to 50%. The FDRs of peptide-spectral matches and proteins were set to <1%.

### Bioinformatics

Functional gene ontology (GO) of analyzed proteins was explicated using the DAVID bioinformatics tool (http://david.abcc.ncifcrif.gov/) and UniprotKB database (http://www.uniprot.org/). GO enrichment analysis for biological process, cellular component, and molecular function, and Kyoto Encyclopedia of Genes and Genomes pathways were generated using Fisher’s exact test (*p*-value < 0.05). The CKD rat kidney data set was uploaded for Ingenuity Pathway Analysis (IPA; Qiagen) for disease and functional analysis. To construct the network model, the protein–protein interactions of proteins that were enriched in fibrosis, apoptosis, and inflammation were obtained from the STRING database (https://string-db.org) and visualized using Cytoscape version 3.7.1 (http://cytoscape.org). For comparative bioinformatics analysis, external proteomic repository, including the Mouse Kidney Fibromics (http://hbcreports.med.harvard.edu/fmm/) (27), was utilized.

### Western Blot

Protein was extracted from the cells using radioimmunoprecipitation assay buffer containing the Halt protease inhibitor (Pierce). Kidneys were harvested 4 and 8 weeks after 5/6 nephrectomy. The kidney tissue was homogenized in PBS containing complete protease inhibitor cocktail (Roche Applied Science). Western immunoblotting was performed using primary antibodies against fibronectin (Abcam), phospho-Ser536-P65 (Cell Signaling Technology), protein S (Proteintech), galectin-1 (Invitrogen), vimentin (Santa Cruz Biotechnology), and β-actin (Sigma-Aldrich). Briefly, equal amounts (40 μg) of the extracted proteins were separated on 10% SDS-polyacrylamide gels and transferred onto Immobilon-FL 0.4 μM polyvinylidene difluoride membranes (Millipore). Anti-rabbit IgG (Cell Signaling Technology) and anti-mouse IgG (Cell Signaling Technology) were used as horseradish-peroxidase-conjugated secondary antibodies. The intensities of the immunoblot band were visualized and captured using an Image Quant LAS 4000 mini (GE Healthcare). The densitometric analysis of the protein expression was quantified using ImageJ (National Institutes of Health, Bethesda, MD, USA).

### Quantitative Reverse-Transcription Polymerase Chain Reaction (qRT-PCR)

Total RNA was extracted from the cells and tissues, and the mRNA levels of the target genes were assayed by qRT- PCR. Briefly, total RNA was extracted from the primary cultured GECs and 5/6 nephrectomized kidney tissues using the RNeasy kit (Qiagen GmbH), and 500 ng of total RNA was reverse-transcribed using oligo-d(T) primers and AMV-RT Taq polymerase (Promega). qRT- PCR was performed using either Assay-on-Demand TaqMan probes or the SYBR Green method and primers for *PROS1*, *LGALS1*, *NFKB1*, *P53*, *FN1*, and *GAPDH* (Applied Biosystems) and analyzed using the Applied Biosystems PRISM 7500 sequence detection system. Data were normalized to *GAPDH* and presented as fold increase compared with RNA isolated from the control group using the 2^−ΔΔCT^ method (28). All experiments were performed in triplicate. PCR primers used for qRT-PCR are listed in supplemental Table S1.

### Immunohistochemistry

Immunohistochemical staining was performed on rat kidney tissues and human kidney biopsy tissues. Human kidney biopsy tissues were obtained from the patients who provided written informed consent (IRB number: 1710-058-894). Periodic acid–Schiff base staining and Masson’s trichrome staining were performed to evaluate the level of fibrosis. For the immunohistochemical assays, paraffin-embedded sections of rat and human kidneys, cut into 4 μm-thick slices, were deparaffinized and hydrated using xylene and ethanol. Endogenous streptavidin activity was blocked using 3% hydrogen peroxide. All kidney sections were prepared in identical fashion. The deparaffinized sections were stained with an anti-PROS-1 antibody (OriGene) and anti-LGALS-1 antibody (Invitrogen) and then incubated with the appropriate fluorophore-linked secondary antibody (goat anti-rabbit IgG). Next, 3,3'-diaminobenzidine tetrahydrochloride (Sigma-Aldrich) was used for immunohistochemical detection. Finally, all sections were counterstained with Mayer’s hematoxylin (Sigma-Aldrich) and evaluated under a light microscope using a camera with differential interference contrast (DFC-295; Leica).

Detailed information of the antibodies used for western blot and immunohistochemistry is provided in supplemental Table S2.

### Statistical Analysis

Perseus software was used for all statistical analyses of MS data (29). In case of label-free quantification, iBAQ intensities were log2-transformed. After filtering out proteins with at least 70% valid values in each group, missing values were imputed assuming a normal distribution of 0.5 width and 1.8 downshifts. The data were then normalized *via* width adjustment, which subtracts the medians and scales all values in a sample to have equal interquartile ranges (30). To compare the four groups of urine samples, we performed analysis of variance (ANOVA) at FDR adjusted *p*-value of 0.05. Normalized protein abundance levels were subjected to further z-normalization followed by hierarchical clustering in terms of the Euclidean distance and average linkage. For pairwise comparison of rat kidney tissue, two-sample *t*-tests were performed at a significance level of permutation-based FDR of 5%. For TMT 6-plex experiments, reporter ion intensities were log2-transformed. After the data were normalized using width adjustment in Perseus software, two-sample *t*-tests were performed using permutation-based FDR and a significance level of 5%.

For validation analysis, statistical analysis was performed using GraphPad Prism 8.3 software (Graph Pad, Inc). Data were expressed as the means ± standard deviation or means ± standard error of the mean, wherever indicated. Pairwise comparison of multiple groups was performed with Kruskal–Wallis test and FDR adjustment. *p*-values < 0.05 were considered statistically significant.

## Results

### Overall Strategy for Profiling Proteins Associated With Chronic Kidney Injury in Urine, Kidney Tissue, and Renal Cells

**Fig. 1:**
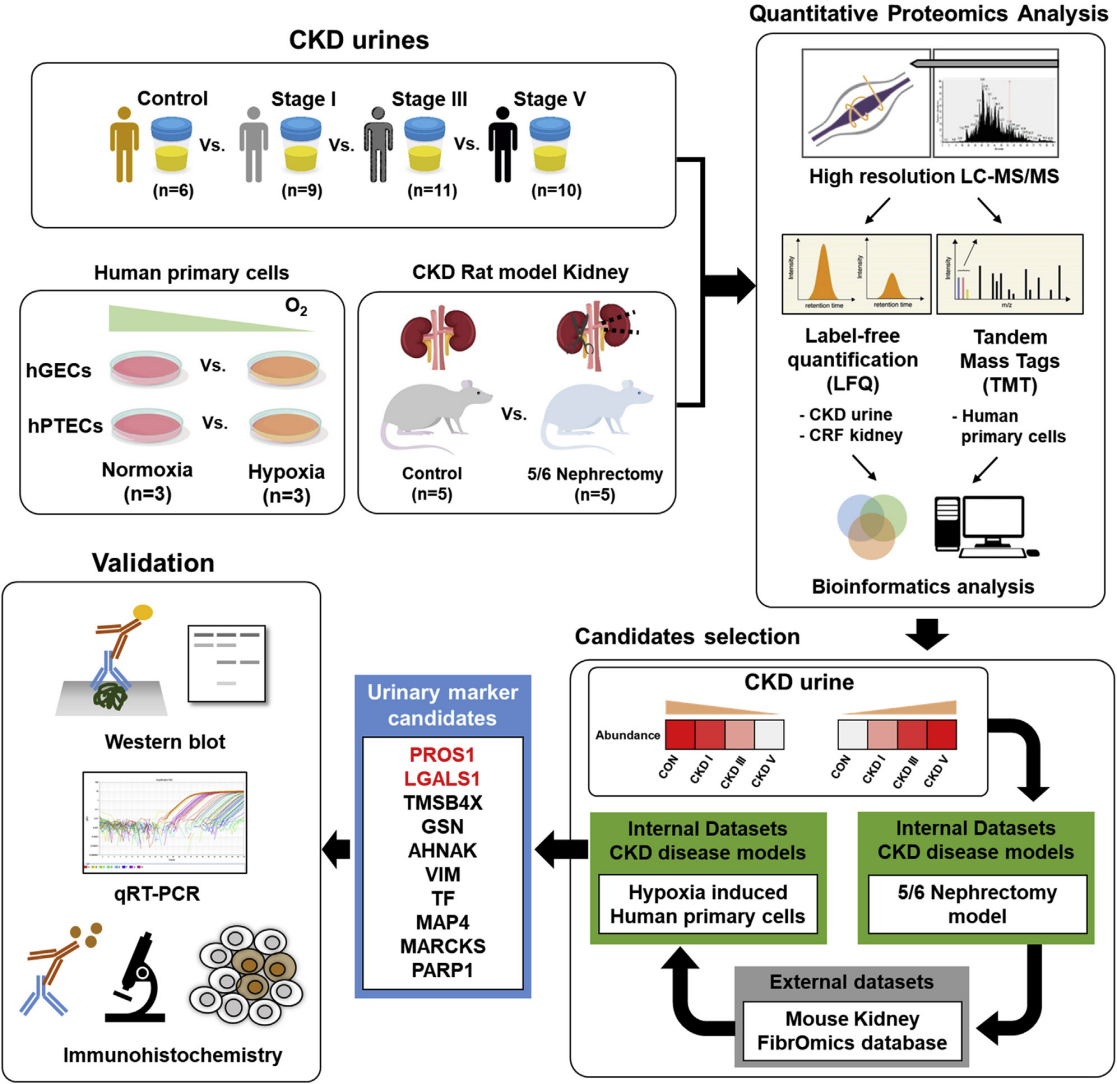
**Overview of the study design.** Briefly, proteomics analysis of the human urine samples was performed, and the cross-linked proteins were identified by proteomics analyses of rat kidney tissue and human primary cultured renal cells. Then, the identified differentially expressed proteins were cross-validated using external databases; finally, the identified proteins were validated using various laboratory methods.

In this study, we developed a workflow to discover markers of parenchymal injury associated with chronic injury. A schematic summary of the overall multistep workflow is presented in Figure 1. Validation was performed through various laboratory methods for specific proteins that repeatedly showed significant differences in various samples.

### Difference of Urine Proteome According to CKD Stage

A total of 36 urine samples from 30 patients with CKD and 6 healthy controls were analyzed by label-free MS. The clinical characteristics of the enrolled participants are listed in Table 1. The mean eGFR of healthy controls and the patients with CKD stages 1, 3, and 5 were 108.1 ± 8.8, 110.1 ± 17.9, 43.2 ± 12.5, and 10.5 ± 3.0 ml/min/1.73 m^2^, respectively; urine protein-to-creatinine ratios were 0.06 ± 0.03, 0.5 ± 0.3, 1.3 ± 1.2, and 3.5 ± 1.7 mg/mg, respectively (Table 1).

**Table 1:** Baseline characteristics of the study participants including the healthy controls and the patients with CKD stages 1, 3, and 5 who provided urine samples for mass spectrometry

Variables	Control N = 6	CKD 1 N = 9	CKD 3 N = 11	CKD 5 N = 10	*p*-value^a^
Age, years	35.3 ± 7.13	34.8 ± 15.5	55.6 ± 11.2	56.7 ± 18.0	0.010
Male sex, number (%)	2 (33.3)	6 (66.7)	4 (36.4)	6 (60.0)	0.351
Blood urea nitrogen, mg/dl	12.8 ± 3.5	13.2 ± 5.4	26.6 ± 7.6	60.9 ± 25.3	<0.001
Creatinine, mg/dl	0.8 ± 0.1	0.8 ± 0.2	1.5 ± 0.4	5.6 ± 1.6	<0.001
estimated GFR, ml/min/1.73 m^2^	108.1 ± 8.8	110.1 ± 17.9	43.2 ± 12.5	10.5 ± 3.0	<0.001
Urine protein/creatinine ratio	0.06 ± 0.03	0.5 ± 0.3	1.3 ± 1.2	3.5 ± 1.7	0.002
Hemoglobin, g/dl	13.4 ± 0.6	14.3 ± 0.9	11.6 ± 1.6	9.8 ± 1.4	<0.001
Albumin, g/dl	4.7 ± 0.1	4.4 ± 0.3	4.2 ± 0.4	3.7 ± 0.6	0.013
Cause of renal dysfunction, number (%)					0.680
IgA nephropathy	NA	4 (44.4)	4 (36.4)	2 (20.0)	
Focal segmental glomerulosclerosis	NA	0 (0)	1 (9.1)	1 (10.0)	
Tubulointerstitial nephritis	NA	0 (0)	2 (18.2)	1 (10.0)	
Hypertension	NA	0 (0)	1 (9.1)	1 (10.0)	
Diabetes mellitus	NA	1 (11.1)	0 (0)	2 (20.0)	
Unknown	NA	5 (55.6)	3 (27.3)	3 (30.0)	

Abbreviations: CKD, chronic kidney disease; GFR, glomerular filtration rate; NA, not available.

a Calculated among CKD groups.

**Fig. 2:**
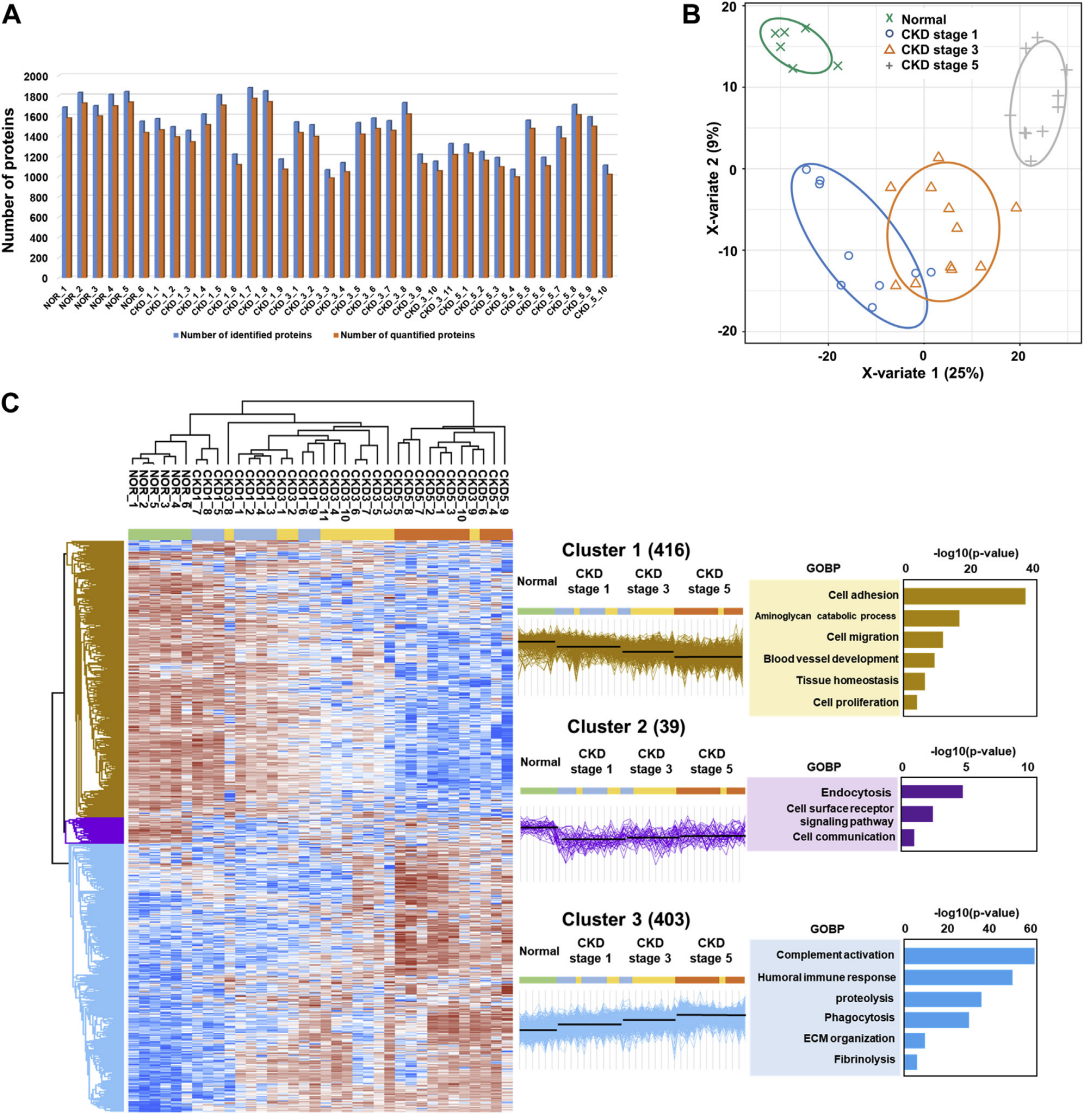
**Identification and classification of the differentially expressed proteins according to chronic kidney disease (CKD) stages in human urine samples by label-free mass spectrometry.***A*, bar charts of the identified (*blue*) and quantified (*red*) proteins in each CKD stage and control urine sample. *B*, PLS-DA score plot of the control and CKD stage 1, 3, and 5 urine proteins. *C*, hierarchical clustering analysis among the urine samples. Three main clusters were identified, and their related gene ontology biological process terms derived from the DAVID functional annotation classification tool with *p*-values  < 0.05 are listed. CKD, chronic kidney disease; GOBP, gene ontology term for biological process.

A total of 2607 unique proteins were identified and quantified among all samples (supplemental Table S3). The average protein number identified in each sample was 1481 (Fig. 2*A*). Following implementation of a normalization process to correct for systematic bias across comparison groups, 858 differentially expressed proteins (DEPs) were identified by ANOVA with permutation-based FDR of 5% among the healthy controls and three groups of CKD urine samples (supplemental Table S4). To reveal changes in proteomics profile according to CKD stage, partial least squares–discriminant analysis (PLS-DA) was performed. The PLS-DA score plot showed that the three groups of CKD urine samples as well as healthy controls were unambiguously divided into four categories, which indicated that urinary proteins clearly changed with increasing CKD severity (Fig. 2*B*).

To define and clarify the expression patterns of DEPs in the healthy controls and the three groups of CKD urine samples, hierarchical clustering was performed, which revealed three different patterns of protein expression change according to the control group and CKD stages. Among the clusters, two clusters with sequentially decreasing or increasing patterns of protein expression according to increasing CKD stage were dominant with 416 and 403 proteins, respectively (Fig. 2*C*). Notably, each cluster in these three proteomic trajectories was associated with different GO terms. In particular, a cluster of proteins that sequentially increased with increasing CKD severity showed significantly enriched GO terms associated with immune and inflammatory responses such as proteolysis, complement activation, humoral immune response, and phagocytosis. In comparison, a cluster that sequentially decreased with increasing CKD severity was significantly enriched in GO terms related to cell adhesion and cell migration, suggesting that factors associated with cell-to-cell junctions were decreased concomitant with renal function deterioration. The enriched GO terms for biological processes of each cluster are shown in Figure 2*C*.

### Comprehensive Identification of Renal Tissue Proteomes Associated With Chronic Renal Injury *via In Vivo* CKD Models With 5/6 Nephrectomy

**Fig. 3:**
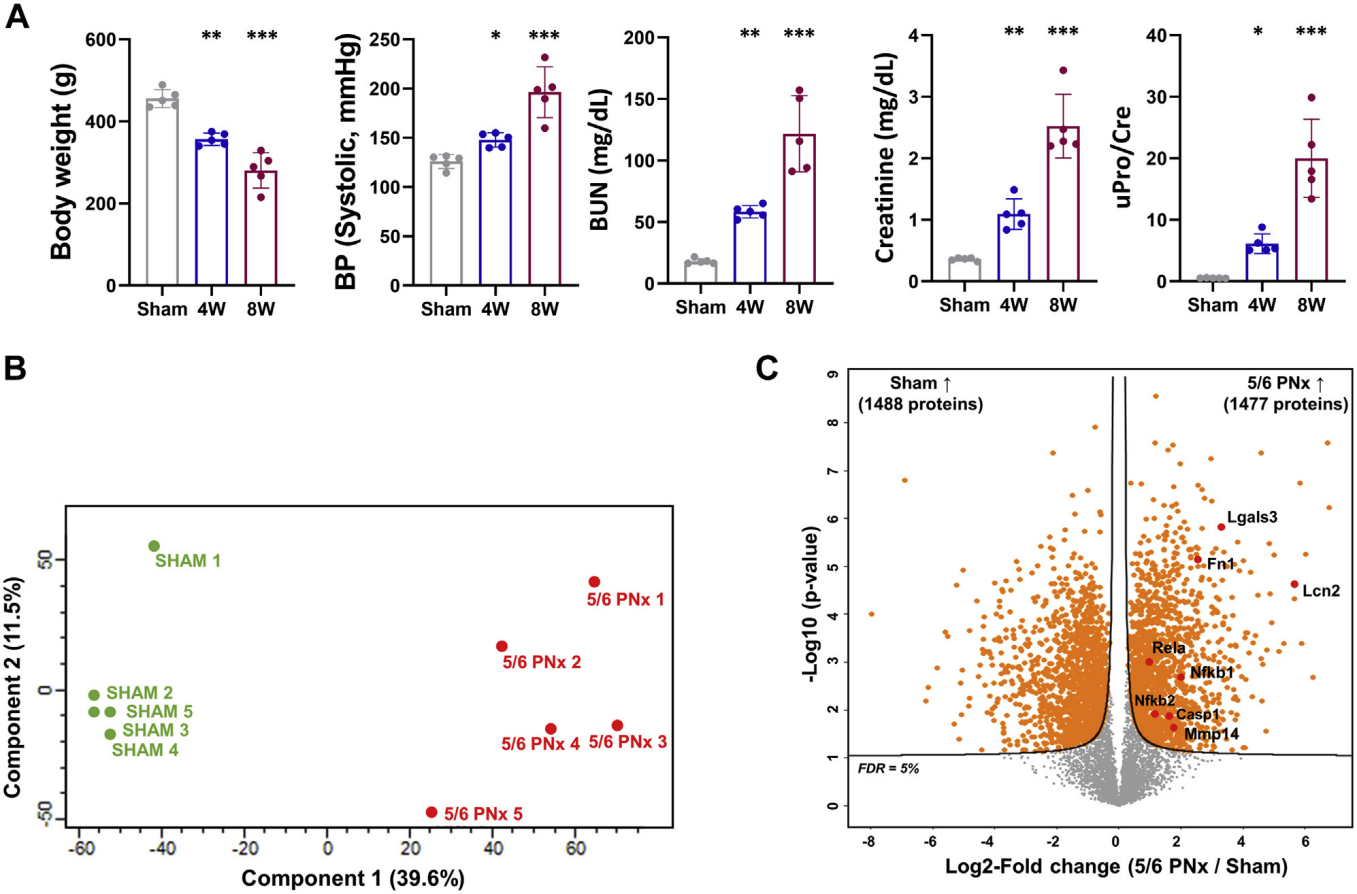
**Identification of the differentially expressed proteins in the rat kidney tissue between 5/6 nephrectomy models and sham-operated controls.***A*, as a chronic kidney disease model, 5/6 nephrectomy was performed and several clinical factors including body weight, blood pressure, blood urea nitrogen (BUN), creatine, and urine protein/creatinine (uPro/Cre) ratio were analyzed after 4 and 8 weeks. *Asterisks* in the densitometry are calculated by comparison with the levels of sham-operated rat kidney tissue. *Gray*, *blue*, and *red colored bar charts* represent sham-operated, 4 weeks after 5/6 nephrectomy, and 8 weeks after 5/6 nephrectomy groups, respectively. Error bars represent standard deviation. ∗*p* < 0.05; ∗∗*p* < 0.005; ∗∗∗*p* < 0.001. *B*, principal component analysis plot of the tissue proteins from the sham-operated controls and 8 weeks after 5/6 nephrectomy using the first two principal components. The plot displays PC1 on the x axis and PC2 on the y axis. *C*, volcano plot depicting the variance in expression between sham-operated control and 5/6 nephrectomized rat kidney tissue. Highlighted data points indicate the already known proteins related to inflammation, apoptosis, and fibrosis processes. The solid line indicates the cutoff based on false discovery rate <5%.

We next conducted tissue proteomics analysis for defining the change of proteins expressed in renal tissue according to chronic renal injury. At first, we induced chronic renal injury in rats through 5/6 nephrectomy. Subsequently, CKD rats exhibited a significant decrease in body weight over time compared with that of the sham-operated group. Blood urea nitrogen and creatinine, which reflect renal function, increased significantly at 4 and 8 weeks after nephrectomy (Blood urea nitrogen at 4 weeks and 8 weeks: 58.4 ± 5.0 and 121.8 ± 30.9 mg/dl; creatinine at 4 weeks and 8 weeks: 1.1 ± 0.2 and 2.5 ± 0.5 mg/dl). In addition, CKD rats demonstrated significantly high levels of proteinuria compared with those of the sham-operated group (0.5 ± 0.0 *versus* 6.4 ± 2.8, *p* < 0.001 at 8 weeks Fig. 3*A*). Rat kidney tissues for MS after 5/6 nephrectomy were harvested at 8 weeks.

We evaluated the proteomes of five kidney tissues each for the CKD and sham-operated groups by label-free MS, identifying and quantifying 8739 and 8704 protein groups, respectively, among all samples (supplemental Table S5). Principal component analysis (PCA) revealed clustering between CKD and sham-operated groups (Fig. 3*B*). Eventually, 2965 DEPs between CKD and sham-operated groups (1477 upregulated and 1488 downregulated proteins in rat CKD kidney) were identified at a permutation-based FDR level of 5% (supplemental Table S6). Among these, several fibrosis-related proteins such as fibronectin and matrix metalloproteinase, inflammatory process-related proteins including galectin-3 and caspase-1, and apoptosis-associated proteins including NF-κB, and LCN2 were concurrently elevated in CKD kidney tissues compared with their levels in sham-operated kidney (Fig. 3*C*).

### Comprehensive Identification of Proteomes Associated With Fibrosis in Primary Renal GECs and PTECs Following Hypoxia-Induced Fibrosis Injury

**Fig. 4:**
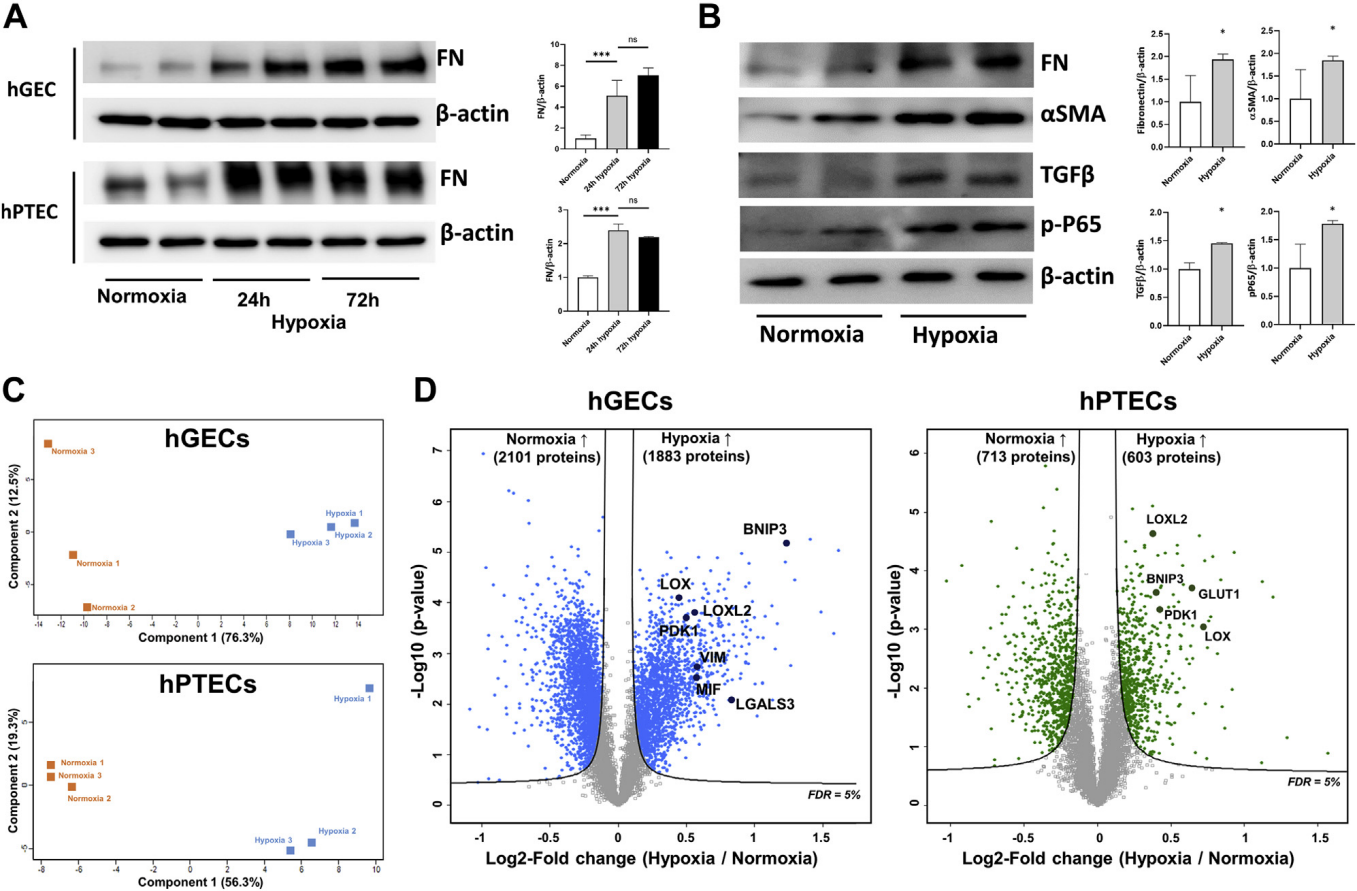
**Identification of the differentially expressed proteins from the human renal cell lysates under normoxic and hypoxic conditions.***A*, fibrosis was induced with a 24-h as well as a 72-h hypoxic injury. A representative blot and densitometry analysis of western blot for fibronectin after hypoxic injury is shown. Error bars represent standard deviation. ns, not significant; ∗∗∗*p* < 0.001. *B*, for the confirmation of chronic injury following hypoxic damage in human GECs, western blot analysis was performed for fibronectin, α-SMA, TGF-β, and phospho-Ser536-P65. A representative blot of three independent experiments is shown. The densitometric analyses of these blots are shown on the right. Error bars represent standard deviation. ∗*p* < 0.05. *C*, principal component analysis plot of the cell-derived proteins from control and hypoxic-injured renal cells using the first two principal components. Upper and lower plots represent principal component analyses for GEC and PTEC, respectively. The plot displays PC1 on the x axis and PC2 on the y axis. *D*, volcano plot depicting the variance in expression between normoxic and hypoxic human primary cultured renal cells. *Left* and *right plots* represent volcano plot for GEC and PTEC, respectively. Marked data points indicate the previously known proteins related to inflammation, apoptosis, and fibrosis processes. The *solid line* indicates the cutoff based on false discovery rate <5%.

To determine the fibrosis-associated change in protein expression at the cellular level, we cultured and isolated primary renal GECs and PTECs (supplemental Fig. S1) and induced fibrosis through hypoxic damage. After 24 h of hypoxia, fibronectin increased significantly compared with its level under normal oxygenation; however, the level of fibronectin showed no difference compared with its level 72 h after hypoxia (Fig. 4*A*). In addition, several other fibrosis markers including α-SMA, TGF-β, and phosphor-Ser536-P65 as well as fibronectin were significantly elevated after 24 h of hypoxia (Fig. 4*B*).

Using a quantitative proteomics approach based on TMT 6-plex, we quantified 7255 and 7112 proteins in GECs and PTECs, respectively (supplemental Table S7). PCA revealed tight clustering in each normoxia and hypoxia sample of GECs and PTECs (Fig. 4*C*). In addition, we identified 3984 and 1321 DEPs under normoxia and hypoxia in GECs and PTECs, respectively, according to permutation-based FDR of 5% (supplemental Table S7). Among these DEPs, various fibrosis-related proteins including LOX, LOXL2, and vimentin along with inflammatory and apoptosis-related proteins including galectin-3, MIF, and BNIF3 were significantly elevated in hypoxic compared with normoxic GECs. Similarly, these fibrosis- and apoptosis-related proteins were also revealed as DEPs in hypoxic and normoxic PTECs (Fig. 4*D*).

### Common Marker Candidates Associated With CKD-Related and Hypoxia-Induced Kidney Injuries

We found significantly increased expression of several proteins associated with inflammation, apoptosis, and fibrosis (supplemental Fig. S2) in our three MS data sets, which represents the sequential process of chronic renal injury.

**Fig. 5:**
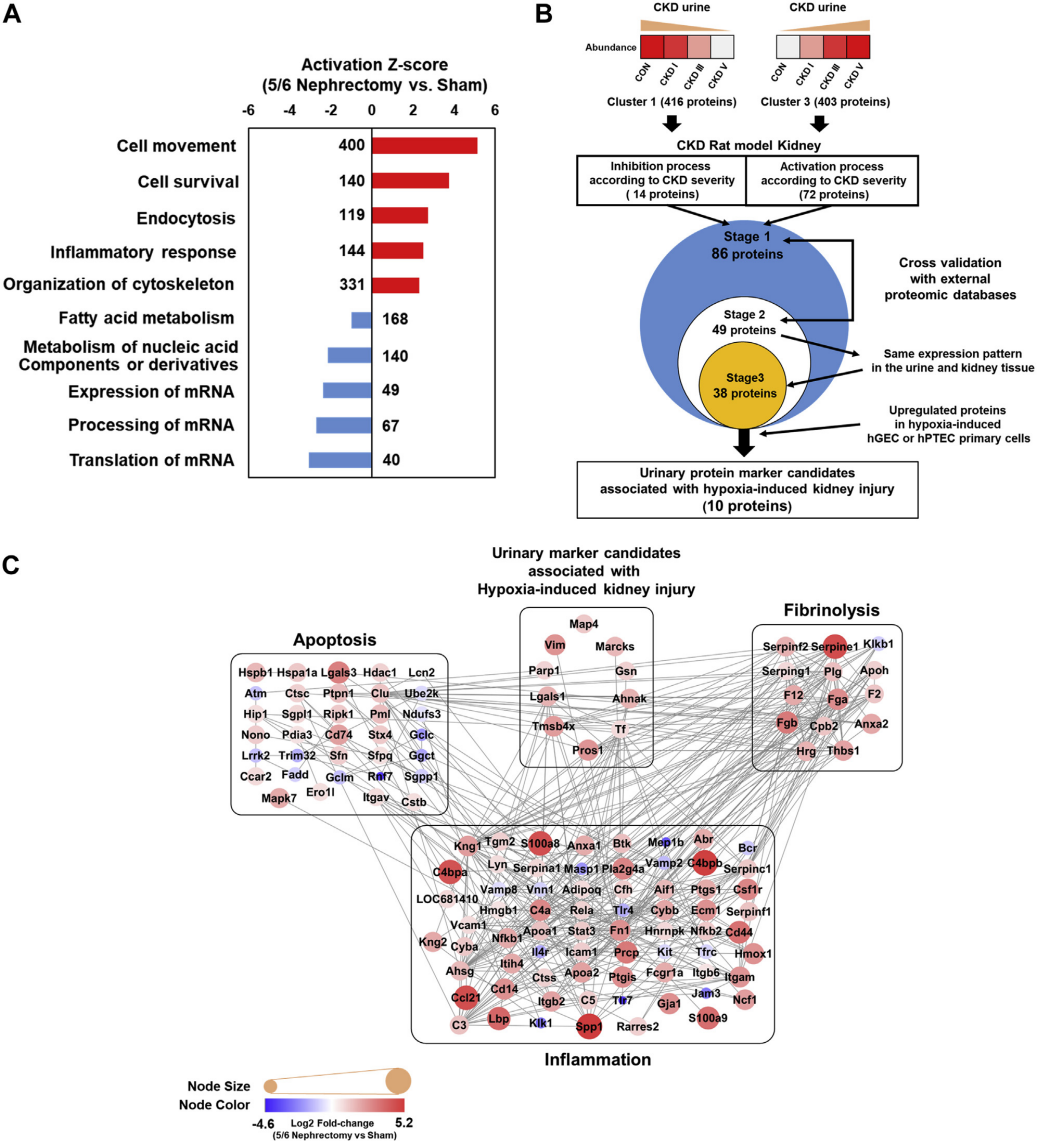
**Protein identification and protein interaction network associated with chronic injury.***A*, ten most differentially expressed proteins (DEPs) between nephrectomized and sham-operated groups of chronic kidney disease (CKD). *B*, schematic flow chart showing sequential analysis along with the number of proteins discovered in each stage of the analysis. Briefly, hierarchical clustered urine DEPs whose levels sequentially increased or decreased with the CKD stages were compared with DEPs identified in CKD rat models to check whether any of these DEPs had same expression patterns in two types of samples (Stage 1). Next, only the significant DEPs were cross validated with external animal experimental data sets (Stage 2) and further shortlisted after comparison of their expression patterns in urine samples (Stage 3). In the final stage, only DEPs that showed same expression patterns in the chronic injury models of human GECs or PTECs were used for further validation analyses. *C*, protein interaction network model related to inflammation, fibrosis, and apoptosis. The overall organization of the protein–protein interaction was visualized using Cytoscape 3.7.1. Color of the inside nodes indicates the expression levels of quantified proteins. *Gray line* represents protein–protein interactions obtained from the STRING database. CKD, chronic kidney disease; DEP, differentially expressed protein; GECs, glomerular endothelial cells; FDR, false discovery rate.

To select urinary marker candidates that are indicative of fibrosis and chronic kidney damage, we first considered candidates from clusters 1 and 3 of the hierarchical clustering that demonstrated change in protein expression level during CKD progression. In the rat CKD kidney dataset, we determined disease-based functional processes according to the CKD severity using the IPA software. As shown in Figure 5*A*, we identified activation of several processes including cell movement, cell survival, endocytosis, inflammatory response, and organization of cytoskeleton in damaged kidneys, while processes including fatty acid metabolism, metabolism of nucleic acids, and regulation of mRNA processing were significantly inhibited (supplemental Table S8). Comparing the urine and rat CKD kidney datasets (supplemental Fig. S3), we identified 86 urinary proteins that were specifically associated with chronic damage in the rat kidneys. Next, 49 urinary proteins were shortlisted according to the expression changes in a reversible chemical-induced injury model (folic-acid-induced nephropathy) or an irreversible surgically induced fibrosis model (unilateral ureteral obstruction) (27) (Fig. 5*B* and supplemental Table S9). Interestingly, well-known kidney injury markers, including N-Gal (LCN2), uromodulin (UMOD), and epidermal growth factor (EGF), were identified in this screening stage, consistent with expression trends that have been reported in previous studies (31, 32, 33, 34). Of the 49 proteins, 38 proteins were identified to have same expression patterns in the urine and CKD kidney tissues according to the CKD severity.

Further, we used data sets of hypoxia-induced primary cell models (GECs or PTECs) for renal fibrosis (35) to identify urinary marker candidates associated with hypoxia-induced kidney injury. Screening of DEPs in hypoxia-induced primary cell models (GECs or PTECs) led to the identification of only ten proteins, including serotransferrin (TF), gelsolin (GSN), poly ADP-ribose polymerase 1 (PARP1), neuroblast differentiation-associated protein AHNAK (AHNAK), microtubule-associated protein 4 (MAP4), galectin-1 (LGALS1), protein S (PROS-1), thymosin beta-4 (TMSB4X), myristoylated alanine-rich C-kinase substrate (MARCKS), and vimentin (VIM) (Table 2 and supplemental Table S9). Thereafter, we analyzed the interaction network models for these ten candidate proteins and found them to be cross-linked in proteomics analyses for all three types of samples. Notably, we found that several proteins associated with inflammation, apoptosis, and fibrosis exhibited significant interactions with these ten candidate proteins (Fig. 5*C*).

**Table 2:** List of six differentially expressed proteins in CKD patient urine samples discovered by mass spectrometry and selected *via* rat tissue and human renal cell proteomic analyses and external validation

Gene names	q-values in ANOVA for 4 urine groups.	Fold change in multiple comparisons of urine samples						Fold change in CKD rat kidney tissue	Fold change after hypoxic damage in hGEC	Fold change after hypoxic damage in hPTEC
		NOR to CKD 1	NOR to CKD 3	NOR to CKD 5	CKD 1–3	CKD 3–5	CKD 1–5			
LGALS-1	6.92E-03	n.s.	6.50	23.99	n.s.	n.s.	16.96	3.364	2.415	0.818
PROS-1	2.03E-06	n.s.	22.04	142.73	4.67	6.48	30.23	5.471	1.784	1.216
TMSB4X	1.14E-02	n.s.	n.s.	44.30	n.s.	n.s.	21.83	4.882	1.845	0.812
TF	1.10E-02	8.148	8.687	4.243	n.s.	n.s.	n.s.	7.95	1.161	n.s.
VIM	3.31E-04	n.s.	n.s.	8.11	3.58	3.30	11.79	2.063	n.s.	1.208
MARCKS	1.70E-03	0.06	n.s.	n.s.	6.09	n.s.	19.81	2.994	n.s.	1.146
PARP1	6.29E-05	9.768	30.425	127.255	n.s.	4.180	13.028	1.913	1.336	n.s.
AHNAK	1.06E-03	n.s.	n.s.	7.035	4.514	n.s.	11.734	3.355	1.422	0.772
GSN	3.31E-04	n.s.	n.s.	8.163	3.596	3.305	11.885	2.063	n.s.	1.208
MAP4	2.21E-03	n.s.	n.s.	19.723	n.s.	8.992	6.859	2.327	1.518	0.885

Abbreviations: CKD, chronic kidney disease; hGEC, human glomerular endothelial cell; hPTEC, human proximal tubular epithelial cell; n.s., not significant; NOR, healthy control with normal renal function.

Fold change of the gene was presented only when FDR <5% was satisfied.

### Validation of Protein Expression

Among the ten significant DEPs, the expression of galectin-1 and protein S, which showed a clear sequential association with CKD stages in urine samples, was further investigated by various methods in renal GECs and rat kidney tissue to confirm the reliability of MS-based protein quantification.

**Fig. 6:**
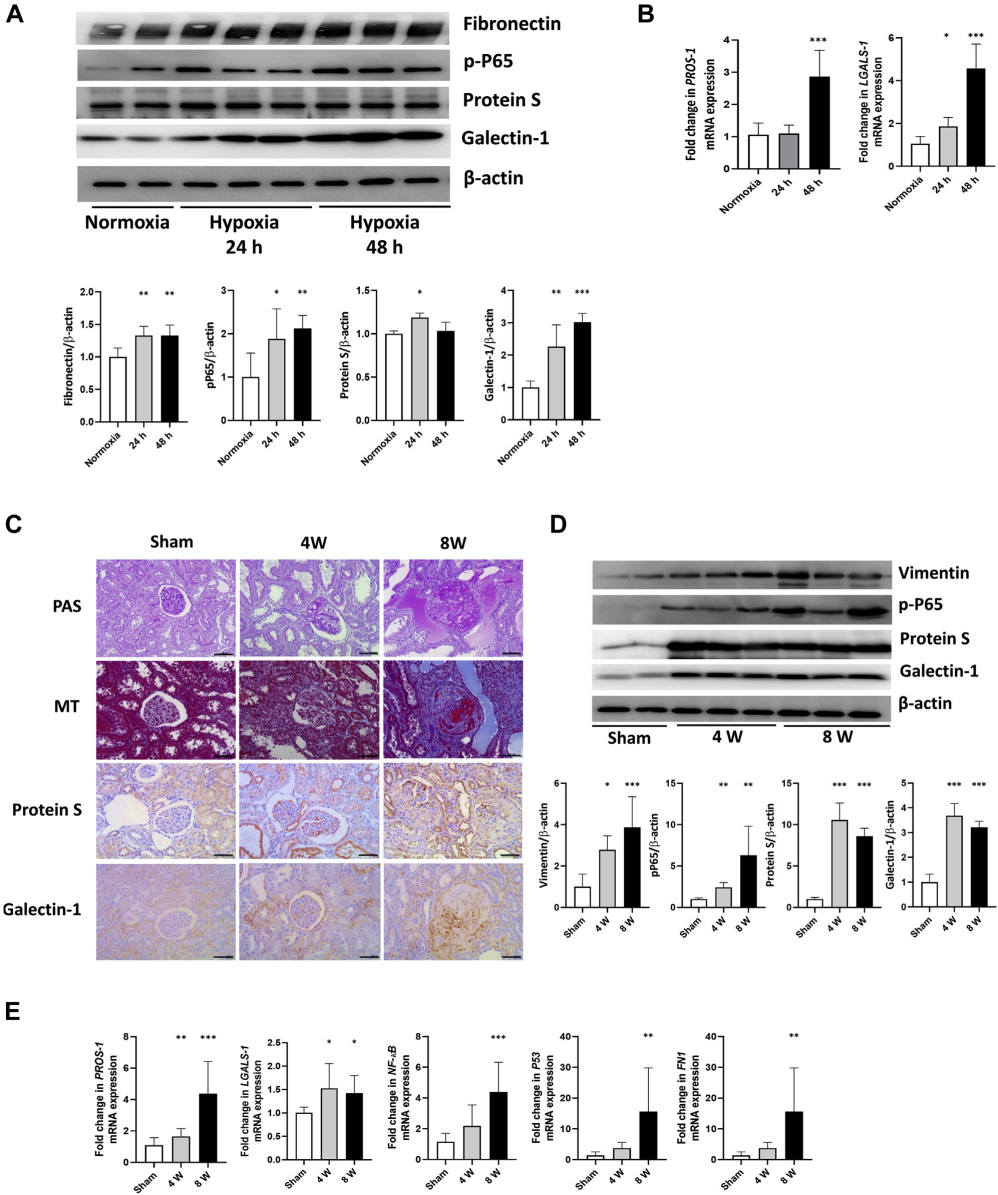
**Validation analysis in human glomerular endothelial cells (GECs) and rat kidney tissues for the identified chronic injury markers, protein S and galectin-1.***A*, western blot analysis was performed for fibronectin, phospho-Ser536-P65, protein S, and galectin-1 at 24 and 48 h after hypoxic injury in GECs. Representative western blot (*upper*) and quantification of the blots (*lower*). *Asterisks* in the densitometry are calculated by comparison with the levels in normoxic GECs. Error bars represent standard deviation. ∗*p* < 0.05; ∗∗*p* < 0.005; ∗∗∗*p* < 0.001. *B*, mRNA expression of PROS-1 and LGALS-1 was measured after 24 and 48 h of hypoxic injury in GECs and compared with the mRNA expression in normoxic GECs. Error bars represent standard deviation. ∗*p* < 0.05; ∗∗∗*p* < 0.001. *C*, periodic acid–Schiff stain, Masson's trichrome stain, and immunohistochemical stain for protein S and galectin-1 of the 5/6 nephrectomized rat kidney tissue at 4 and 8 weeks after injury. Original magnification, ×200. Scale bars: 100 μm. *D*, western blot analysis was performed for vimentin, phospho-Ser536-P65, protein S, and galectin-1 in rat kidney tissues at 4 and 8 weeks after 5/6 nephrectomy. *Asterisks* in the densitometry are calculated by comparison with the levels of sham-operated rat kidney tissue. Error bars represent standard deviation. ∗*p* < 0.05; ∗∗*p* < 0.005; ∗∗∗*p* < 0.001. *E*, *PROS-1*, *LGALS-1*, *NFKB1*, *P53*, and fibronectin mRNA levels were measured in sham-operated rat kidney and 5/6 nephrectomized rat kidney at 4 and 8 weeks. Error bars represent standard deviation. ∗*p* < 0.05; ∗∗*p* < 0.005; ∗∗∗*p* < 0.001.

In the chronic injury model induced through hypoxic damage to renal cells, the well-known fibrosis and apoptosis markers fibronectin and phospho-Ser536-P65 were significantly increased at 24 h of hypoxia and continued to increase at 48 h of hypoxia. Protein S was significantly increased after 24 h of hypoxic damage, whereas at 48 h of hypoxia, the expression level was similar to that before injury. Galectin-1 significantly increased at 24 h after injury and further increased at 48 h (Fig. 6*A*). mRNA expression of both protein S (PROS-1) and galectin-1 (LGALS-1) increased significantly at 48 h (Fig. 6*B*).

In comparison, the 5/6 nephrectomy model revealed increased expression of protein S and galectin-1 at 4 and 8 weeks after nephrectomy in rat tissues. At 4 weeks after injury, protein S and galectin-1 showed similarly increased expression in renal tissue. However, as injury progressed at 8 weeks, protein S was mainly found in the tubulointerstitium rather than glomeruli and galectin-1 was highly expressed in the glomerulus along with sclerotic changes (Fig. 6*C*). Protein and mRNA expression of protein S and galectin-1 was significantly increased from week 4 until week 8 (Fig. 6, *D* and *E*).

**Fig. 7:**
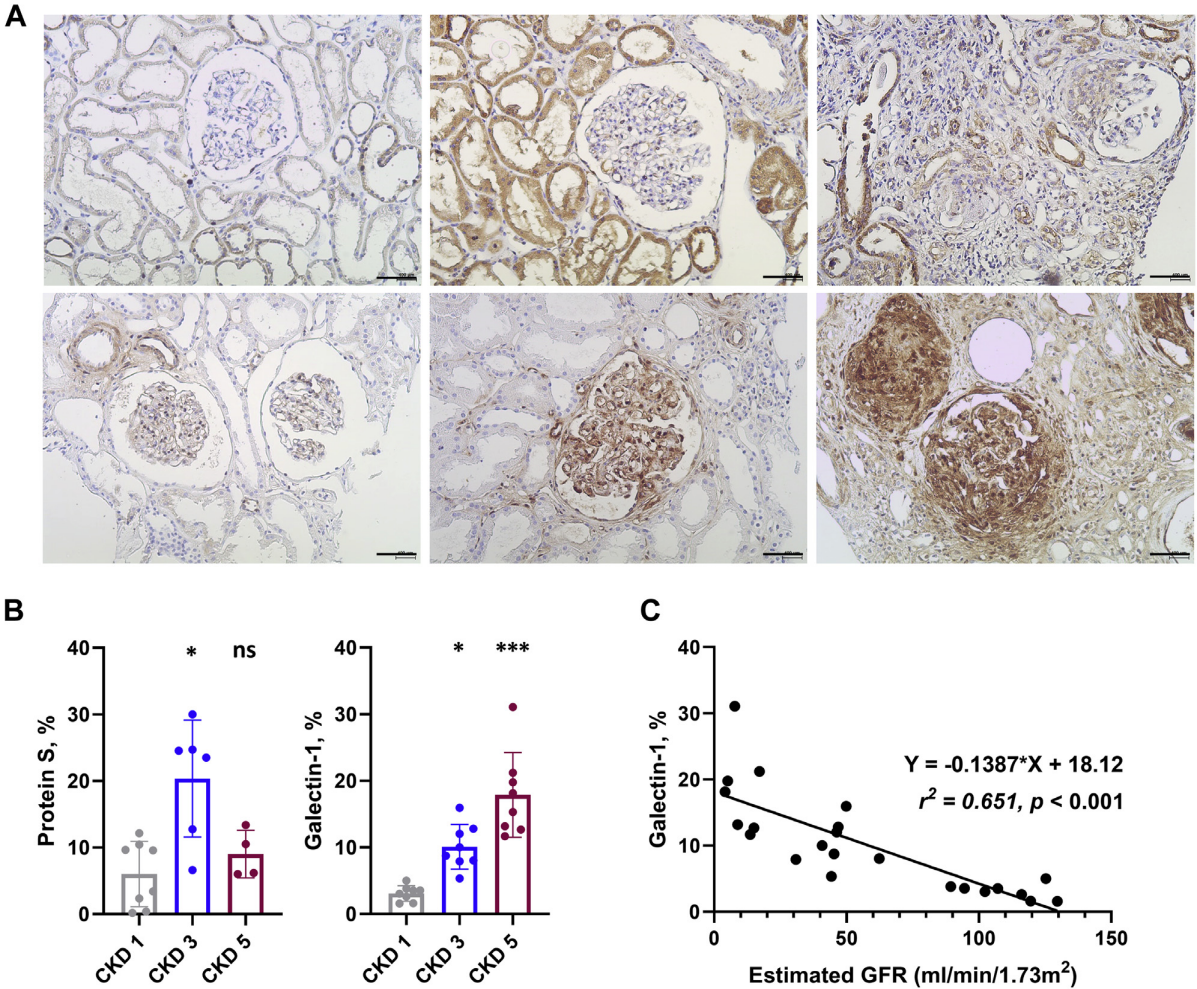
**Validation analysis in renal tissues of patients with CKD stages 1, 3, and 5 for the identified chronic injury markers, protein S and galectin-1.***A*, immunohistochemical stains of protein S (first row) and galectin-1 (second row) in renal tissues of patients with CKD 1, 3, and 5. Original magnification, ×400. Scale bars: 400 μm. *B*, quantification of protein S and galectin-1-positive cells per field. N = 3 fields per slide were evaluated. *Asterisks* in plots were obtained from comparison with the levels in CKD 1. Error bars represent standard deviation. ns, not significant; ∗*p* < 0.05; ∗∗∗*p* < 0.001. *C*, simple linear regression analysis summarizes the relationship between estimated glomerular filtration rate (eGFR) and % of galectin-1 on a scatterplot by fitting a least squares regression line to the data. The regression equation, coefficient of determinant (*r*^*2*^), and *p*-value for the slope of the line are also included in the data field.

Finally, we assessed the expression of protein S and galectin-1 in human kidney tissues (Fig. 7, *A* and *B*) obtained from a different patient cohort than that providing the urine samples used for MS. The expression of protein S was increased at CKD 3 and then decreased at CKD 5, similar to expression patterns in rat tissues, and was mainly found in the tubule rather than the glomerulus. In comparison, the expression of galectin-1 was significantly increased with increasing CKD stage (CKD stage 1, 3 and 5; 3.0 ± 1.2, 10.1 ± 3.4 and 17.9 ± 6.4%, respectively), especially in the glomerular area with sclerotic change. In addition, galectin-1 expression showed significant negative correlation with the eGFR of patients (Regression equation: Y = −0.1387∗X + 18.12, Fig. 7*C*).

## Discussion

CKD is caused by various different factors including diabetes mellitus, hypertension, and glomerulonephritis (36, 37). Because of the associated nephron injury, the development of tubulointerstitial hypoxia, inflammation, oxidative stress, and apoptosis forms a vicious cycle in CKD progression (38, 39, 40). Hypoxia, inflammation, and oxidative stress promote the trans-differentiation of resident fibroblasts, renal erythropoietin-producing cells, or pericytes to extracellular matrix (ECM)-producing myofibroblasts, ultimately leading to renal failure with exacerbation of global fibrosis (39, 41, 42). To identify proteins or genes involved in CKD progression and their pathophysiology, numerous researchers have endeavored to identify and regulate the expression of specific targeted proteins in various renal disease models (31, 43, 44, 45, 46). However, the exact mechanisms of CKD pathophysiology remain to be fully elucidated.

Recently, in the field of kidney disease, attempts to uncover important unknown proteins through untargeted proteomics have been increasing (47). The presence of several proteins in urine or changes in their levels have been proposed as noninvasive urinary biomarkers for CKD in numerous populations (4, 48, 49, 50, 51, 52). In 2010, a human urinary peptidome panel, composed of 273 CKD-specific biomarkers, was identified using capillary electrophoresis-MS (4). The other studies also primarily focused on finding novel biomarkers in various renal diseases (48, 49, 50, 51). In contrast, here, we conducted unsupervised nontargeted MS analysis to identify unknown key functional proteins expressed in the kidney after chronic injury. In the present study, the results of three different proteomics analyses using three types of samples including cells, tissues, and urine identified proteins that exhibited common expression changes following chronic injury by various causes. Furthermore, we revealed interactions between the discovered proteins and other well-known proteins previously shown to be associated with inflammation, apoptosis, and fibrosis.

With the external validation, we observed consistent expression patterns for well-known kidney injury markers, such as N-Gal, uromodulin, and EGF, indicating that our assessment strategy successfully reflected chronic kidney injury as in previous studies. Despite the large number of proteins discovered in each MS-based analysis, only a few specific proteins exhibited same expression patterns in the various types of samples from internal and external validation data sets. Therefore, it is suggested that these proteins may be inclusive markers encompassing heterogeneous types of chronic kidney injuries. Of the ten identified proteins, thymosin beta-4, myristoylated alanine-rich C-kinase substrate, microtubule-associated protein 4, and vimentin play important roles in maintaining cell structure by modulating actin filaments or by acting as a cytoskeleton (53, 54, 55, 56). Furthermore, several reports exist of the association of these proteins with fibrosis in kidney disease (57, 58, 59, 60). Of the remaining proteins, which have rarely been reported with regard to their function in renal disease (61, 62), we conducted validation experiments on two proteins, protein S and galectin-1, to confirm their potential status as candidates in renal pathogenesis.

Protein S and galectin-1 exhibited similar trends of increased expression following chronic renal injury in proteomics analysis using MS, albeit slightly different expression patterns in the validation analysis evaluating various periods following induction of renal injury. In renal cells and human renal tissues, protein S increased in the early phase after injury and decreased in the late phase, whereas galectin-1 increased in the early phase and continued until the late phase of injury. In previous studies, protein S has been shown to be a negative regulator of immune and inflammatory responses and involved in the clearance of apoptotic cells by binding to phosphatidylserine residues exposed early in apoptosis on the surface of apoptotic cells (63, 64, 65, 66). Alternatively, galectin-1 has been related to ECM assembly and remodeling by binding to a number of ECM components in a dose- and β-galactoside-dependent manner and is involved in anti-inflammatory and antiapoptotic processes in various cell types (67, 68). Taken together, these findings suggest that protein S may be predominantly involved in inflammation and apoptosis in the early phase of chronic injury, whereas galectin-1 may participate in the late phase of injury including fibrosis with ECM remodeling in addition to the inflammatory process in chronic injury. These differences in protein function with regard to regulating injury likely underlie the differential expression of these two proteins according to time point in chronic renal injury.

In addition, immunohistochemistry of the rat and human kidney tissue revealed that protein S was mainly expressed in tubulointerstitial lesions, whereas galectin-1 was mainly expressed in the glomerulus. Moreover, the expression of galectin-1 was more significantly changed compared with that of protein S in human GECs. Thus, we considered that these two proteins were expressed in different regions within the renal structure. Although both proteins exhibited similar trends in MS using CKD urine samples and protein S showed higher fold change than did galectin-1 in kidney tissue and urine MS analyses, only galectin-1 demonstrated a significant correlation with eGFR in the validation cohort, suggesting that galectin-1 may have a key role in the chronic injury process of the glomerulus.

Nevertheless, the clinical implications of these proteins need to be further considered. As identified in this study, expressions of protein S and galectin-1 were related to pathways involved in cell and tissue damage and could play significant roles in other diseases. Until now, protein S expression has been studied in oral squamous cell carcinoma and prostate cancer, and galectin-1 expression has been identified in renal clear cell carcinoma. Therefore, protein S and galectin-1 can be considered as the sensitive markers of renal parenchymal injuries; however, further studies are warranted for evaluating the disease specificities of these proteins. Furthermore, the prognostic value of these proteins in CKD progression should be clarified with functional studies.

An advantage of the present study is the use of multiple data sets allowing the comprehensive analysis of the three MS-based proteomics evaluations in human cells, urine, and rat kidney tissue. However, an important limitation of our study is that CKD constitutes a heterogeneous disease group. Because various underlying diseases such as diabetes, hypertension, and glomerulonephritis can cause CKD, the pathophysiology of disease progression in the early phase of CKD might differ according to etiology. Nevertheless, the progression of inflammation, apoptosis, and fibrosis can be considered as a common pathophysiology in advanced CKD. Therefore, although the proteins identified in our study might not be appropriate to reflect the initial changes in each CKD etiology, they may strongly correlate with common mechanisms of the chronic injury in advanced CKD. Similarly, the application of specific injury models for mimicking chronic kidney disease in rat tissues and human cells can be a limitation of our study. Because of the pathologic heterogeneity of CKD, these injury models including hypoxia for cells and 5/6 nephrectomy for rats might not wholly represent the whole pathway of injury in CKD. We tried to address this concern by applying external validation data sets from previous studies that induced various models of kidney disease. Finally, we induced fibrosis in renal cells with 24 h of hypoxia, which is shorter induction duration compared with that used in previous studies. Thus, the chronic injury in renal cells in the present study might represent the early phase of chronic change but not the late phase of fibrosis, even though the fibrosis marker was elevated at as high as 72 h of hypoxia.

The series of inflammatory, apoptotic, and fibrosis processes associated with the irreversible pathologic changes in CKD constitutes an inevitable response to progression to end-stage renal disease; however, many aspects of their initiation and progression have not yet been identified. To our knowledge, this represents the first report of a multisample proteomics study with the aim of identifying key proteins expressed in the kidney that are associated with the various stages of chronic renal damage. The target urinary proteins identified in this study are suitable as markers of chronic damage of renal cells and tissues and may be potential candidates for future research of CKD pathophysiology.

## Data Availability

MS-based proteomics data of all identified peptides and the protein list have been deposited in the ProteomeXchance Consortium (http://proteomecentral.proteomexchange.org) *via* the PRIDE partner repository: data set identifier PXD016433 (CKD urine), PXD016447 (CKD rat kidney), and PXD019678 (hypoxia-induced primary kidney cells).

## Conflict of interest

The authors confirm that there are no conflicts of interest.
