# Supplementary material for: Multisample Mass Spectrometry-Based Approach for Discovering Injury Markers in Chronic Kidney Disease
Source: Mol Cell Proteomics. 2021 Jan 13;20:100037. doi: 10.1074/mcp.RA120.002159 (PMC7950200; doi:10.1074/mcp.RA120.002159)
Supplement: Supplemental Table S1 [file mmc1.docx]

Supplementary material

**Multi-sample mass spectrometry-based approach for discovering injury markers in chronic kidney disease**

Ji Eun Kim^1,*^, Dohyun Han^2,9*^, Jin Seon Jeong^3^, Jong Joo Moon^1^, Hyun Kyung Moon^1^, Sunhwa Lee^4^, Yong Chul Kim^1^, Kyung Don Yoo^5^, Jae Wook Lee^6^, Dong Ki Kim^1,7^, Young Joo Kwon^8^, Yon Su Kim^1,7^, and Seung Hee Yang^7,9^

Supplementary Table S1. Oligonucleotides for RT-PCR

| Transcript | Forward primer (5’-3’) | Reverse primer (5’-3’) |
| --- | --- | --- |
| PROS1 (Rat) | CACAACTGGAGGCAATGTTATT | CACGGCCTCCTTAGCTATTT |
| LGALS1 (Rat) | ACAACCTGTGCCTACACTTC | CAGTCTCCCGTTGTTCTGTT |
| Fibronectin (Rat) | CCAAGTACATTCTCAGGTGGAG | GGTCAGGCCTTTGATGGTATAG |
| NF-κB (Rat) | GGTTACGGGAGATGTGAAGATG | GTGGATGATGGCTAAGTGTAGG |
| P53 (Rat) | GCCGACCTATCCTTACCATC | CTTCTTCTGTACGGCGGTCT |
| GAPDH (Rat) | TGATTCTACCCACGGCAAGTT | TGATGGGTTTCCCATTGATGA |
| PROS1 (human) | GAAGCGTCGTGCAAATTCTTTA | CCTCCCTGGCTTCTTCTTTATT |
| LGALS1 (human) | CCTGAATCTCAAACCTGGAGAG | CACAGGTTGTTGCTGTCTTTG |
