# Supplementary material for: Multisample Mass Spectrometry-Based Approach for Discovering Injury Markers in Chronic Kidney Disease
Source: Mol Cell Proteomics. 2021 Jan 13;20:100037. doi: 10.1074/mcp.RA120.002159 (PMC7950200; doi:10.1074/mcp.RA120.002159)
Supplement: Supplemental Table S2 [file mmc2.docx]

Supplementary material

**Multi-sample mass spectrometry-based approach for discovering injury markers in chronic kidney disease**

Ji Eun Kim^1,*^, Dohyun Han^2,9*^, Jin Seon Jeong^3^, Jong Joo Moon^1^, Hyun Kyung Moon^1^, Sunhwa Lee^4^, Yong Chul Kim^1^, Kyung Don Yoo^5^, Jae Wook Lee^6^, Dong Ki Kim^1,7^, Young Joo Kwon^8^, Yon Su Kim^1,7^, and Seung Hee Yang^7,9^

Supplementary Table S2. Antibodies used for western blotting and immunohistochemistry.

| Antibodies | Vendor | catalog number | dilution |
| --- | --- | --- | --- |
| Western blot |  |  |  |
| fibronectin (human) | Santa Cruz Biotechnology, Dallas, TX, USA | sc-8422 | 1/300 |
| αSMA (human) | Abcam, Cambridge, MA, USA | ab7817 | 1/400 |
| TGFβ (human) | Abcam, Cambridge, MA, USA | ab66043 | 1/300 |
| phospho-Ser536-P65 (human) | Cell Signaling Technology, Danvers, MA, USA | 3033L | 1/500 |
| β-actin (human) | Sigma-Aldrich, , St. Louis, MO, USA | A1978 | 1/10000 |
| PROS-1 (human) | Proteintech, Chicago, IL, USA | 16910-1-AP | 1/500 |
| LGALS-1 (human) | Invitrogen, Carlsbad, CA, USA | MA5-32779 | 1/500 |
| vimentin (rat) | Santa Cruz Biotechnology, Dallas, TX, USA | sc-7558 | 1/100 |
| phospho-Ser536-P65 (rat) | Cell Signaling Technology, Danvers, MA, USA | 3033L | 1/500 |
| PROS-1 (rat) | Proteintech, Chicago, IL, USA | 16910-1-AP | 1/500 |
| LGALS-1 (rat) | Invitrogen, Carlsbad, CA, USA | MA5-32779 | 1/500 |
| β-actin (rat) | Sigma-Aldrich, , St. Louis, MO, USA | A1978 | 1/10000 |
| Immunohistochemistry |  |  |  |
| PROS-1 (rat) | OriGene, Rockville, MD, USA | TA321108 | 1/100 |
| LGALS-1 (rat) | Invitrogen, Carlsbad, CA, USA | MA5-32779 | 1/200 |
| PROS-1 (human) | OriGene, Rockville, MD, USA | TA321108 | 1/100 |
