# Supplementary material for: Multisample Mass Spectrometry-Based Approach for Discovering Injury Markers in Chronic Kidney Disease
Source: Mol Cell Proteomics. 2021 Jan 13;20:100037. doi: 10.1074/mcp.RA120.002159 (PMC7950200; doi:10.1074/mcp.RA120.002159)
Supplement: Supplemental Figure S1 [file mmc9.pptx]

## Slide 1
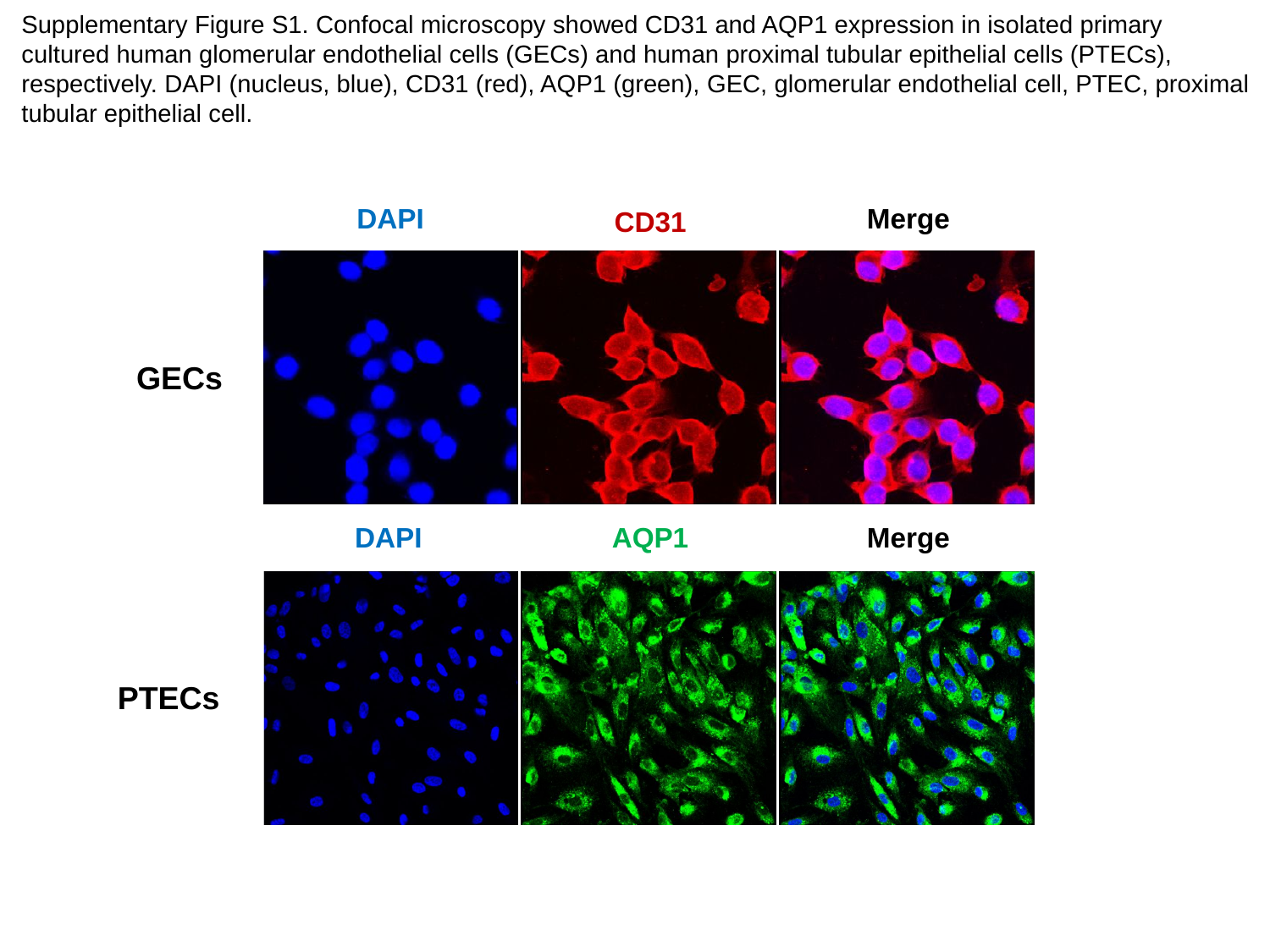

Supplementary Figure S1. Confocal microscopy showed CD31 and AQP1 expression in isolated primary cultured human glomerular endothelial cells (GECs) and human proximal tubular epithelial cells (PTECs), respectively. DAPI (nucleus, blue), CD31 (red), AQP1 (green), GEC, glomerular endothelial cell, PTEC, proximal tubular epithelial cell.
Merge
DAPI
CD31
GECs
Merge
DAPI
AQP1
PTECs

## Slide 2
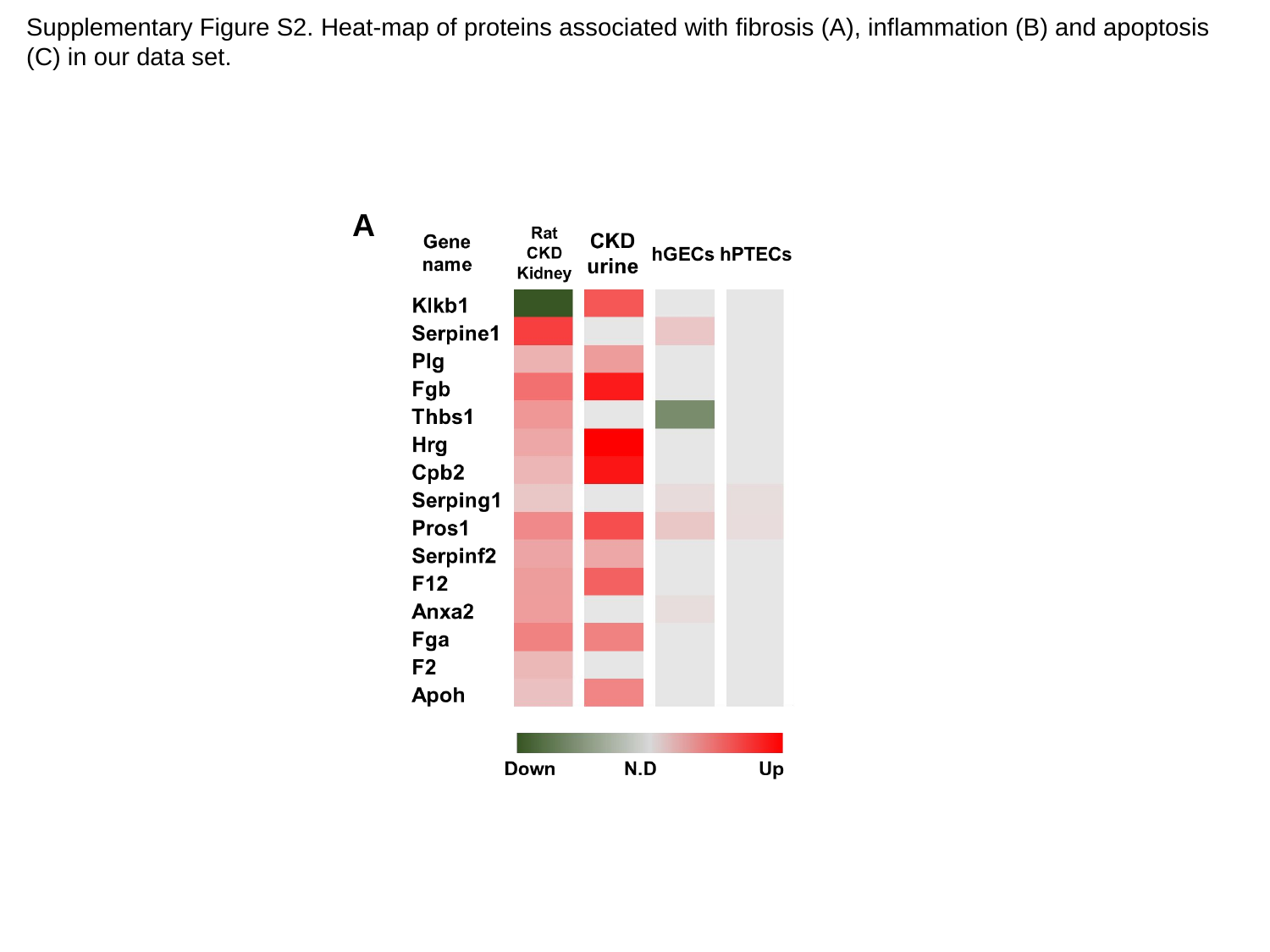

Supplementary Figure S2. Heat-map of proteins associated with fibrosis (A), inflammation (B) and apoptosis (C) in our data set.
A

## Slide 3
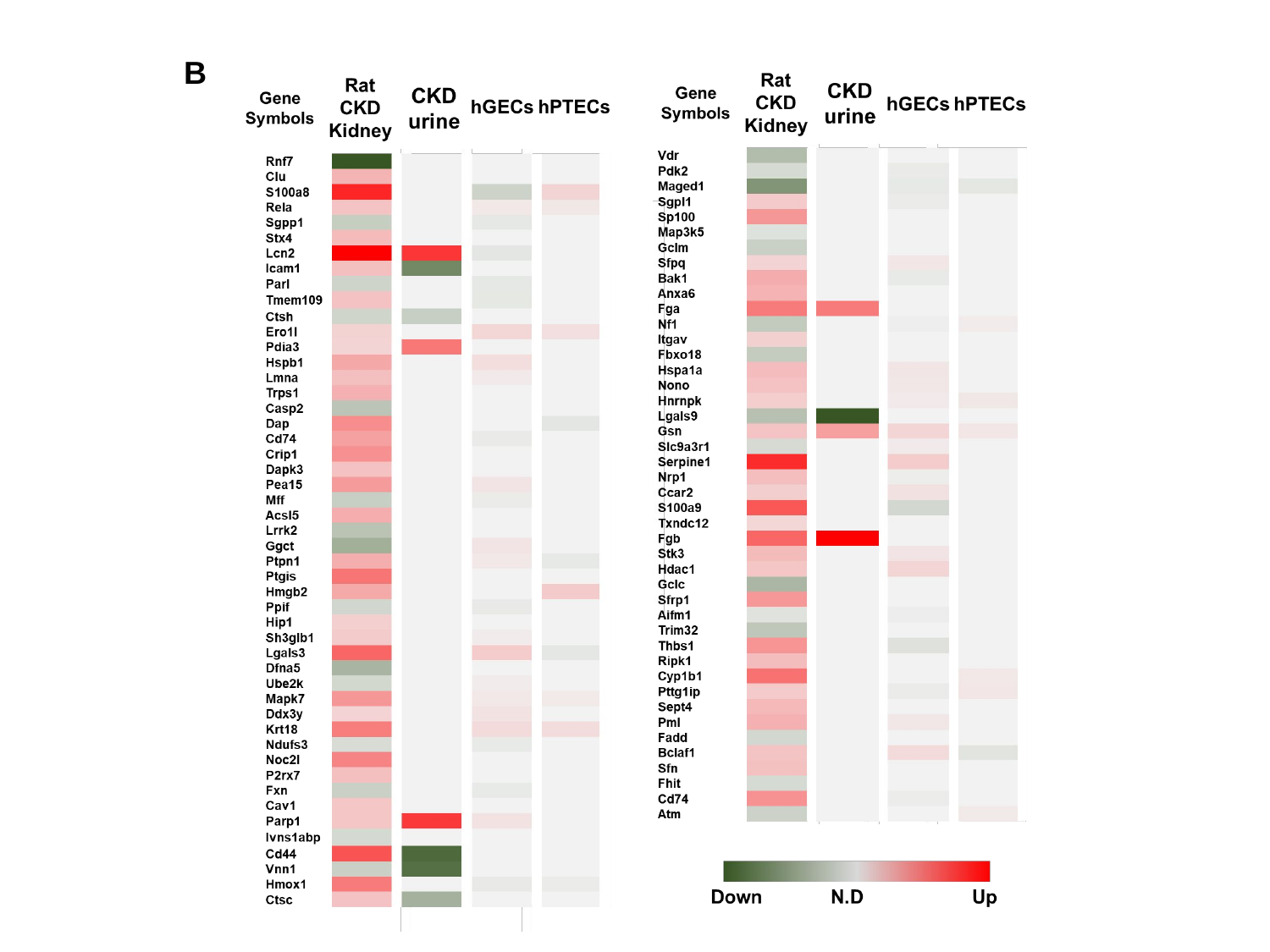

B

## Slide 4
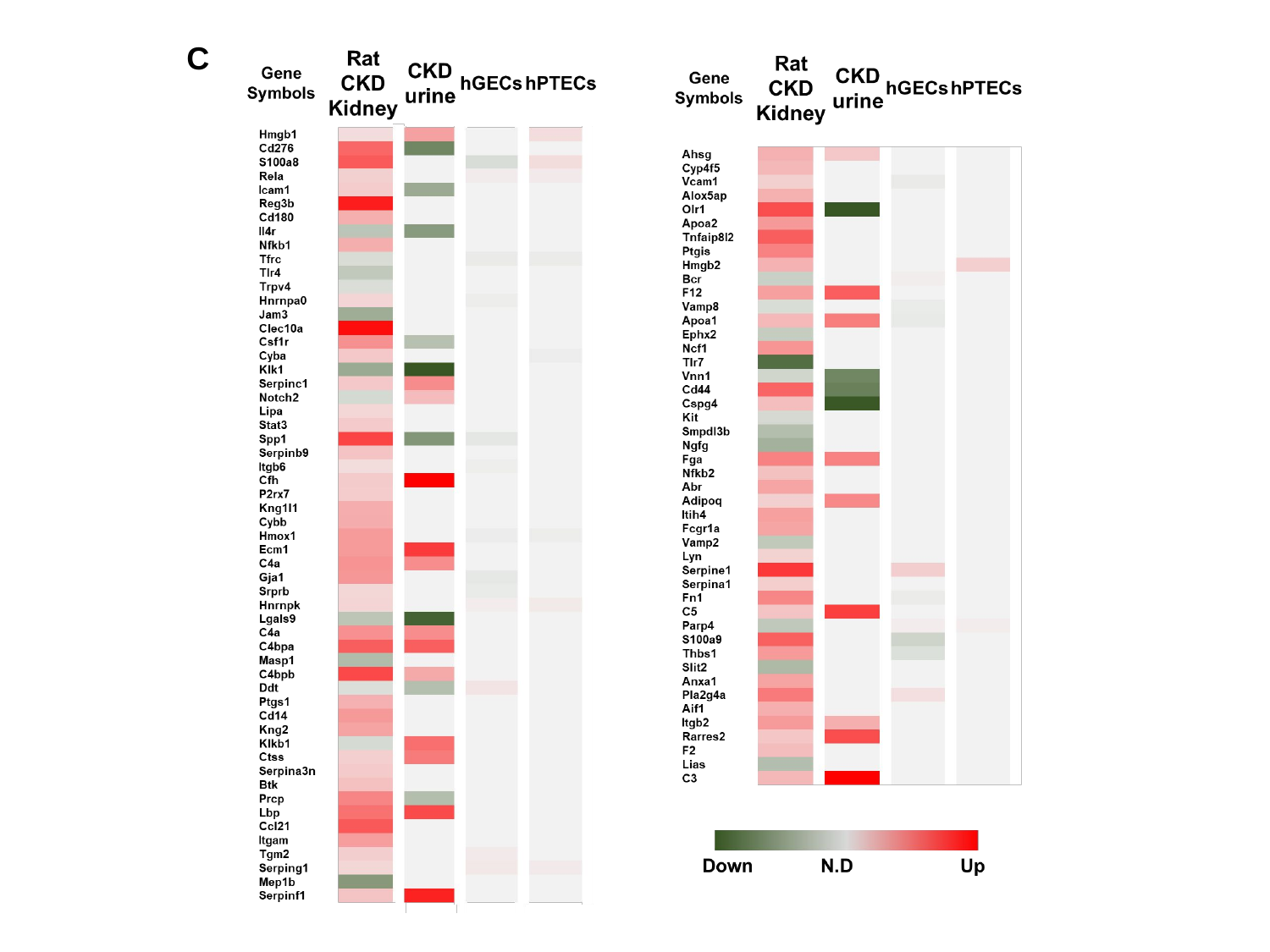

C

## Slide 5
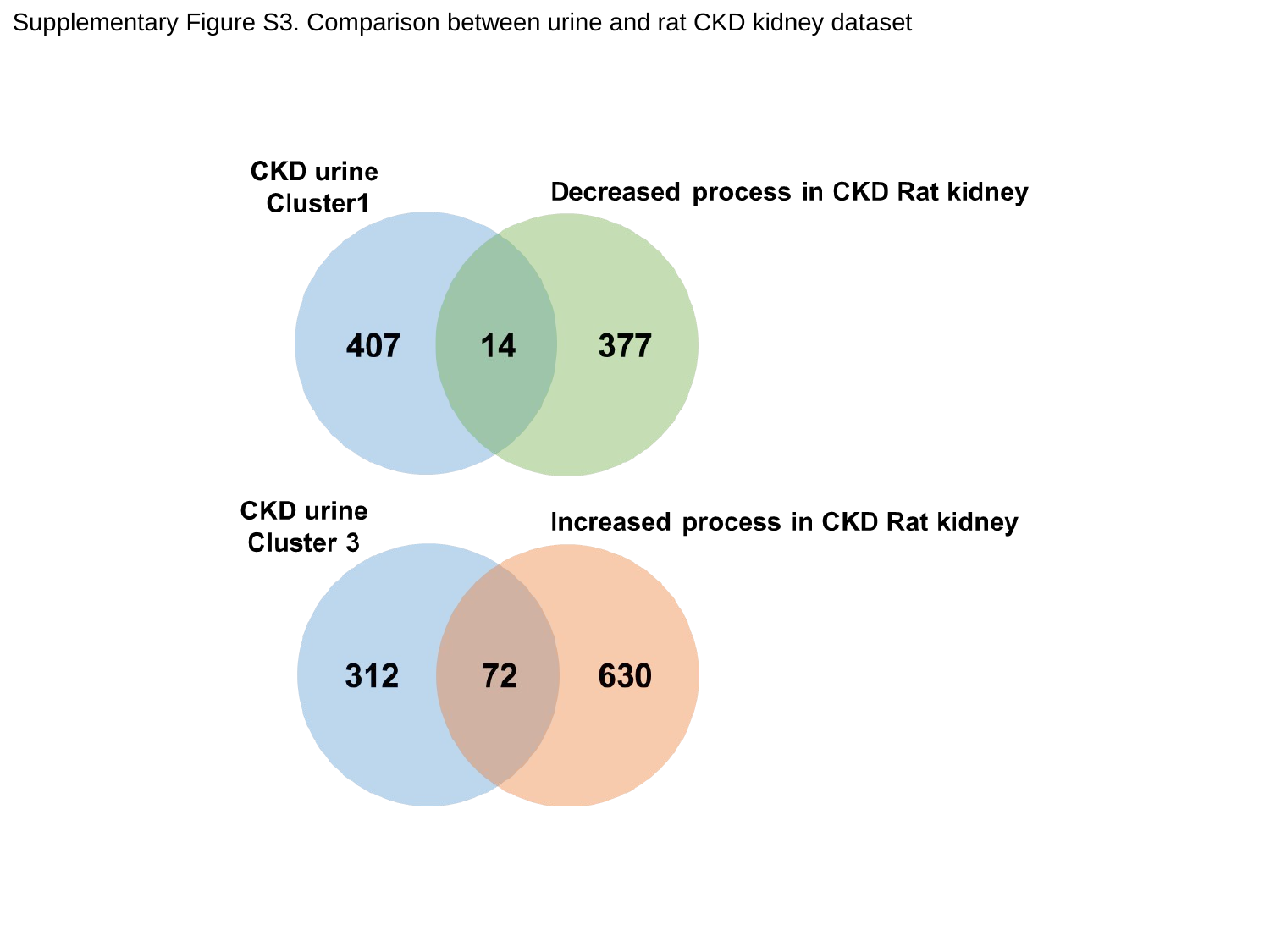

Supplementary Figure S3. Comparison between urine and rat CKD kidney dataset
